# Hydroxyapatite and Other Calcium Phosphates for the Conservation of Cultural Heritage: A Review

**DOI:** 10.3390/ma11040557

**Published:** 2018-04-04

**Authors:** Enrico Sassoni

**Affiliations:** Department of Civil, Chemical, Environmental and Materials Engineering (DICAM), University of Bologna, Via Terracini 28, 40131 Bologna, Italy; enrico.sassoni2@unibo.it; Tel.: +39-051-2090329

**Keywords:** marble, limestone, consolidation, protection, durability, calcium phosphates, hydroxyapatite, octacalcium phosphate, ammonium phosphate, ammonium oxalate

## Abstract

The present paper reviews the methods and the performance of *in situ* formation of calcium phosphates (CaP) for the conservation of materials belonging to cultural heritage. The core idea is to form CaP (ideally hydroxyapatite, HAP, the most stable CaP at pH > 4) by reaction between the substrate and an aqueous solution of a phosphate salt. Initially proposed for the conservation of marble and limestone, the treatment has been explored for a variety of different substrates, including sandstones, sulphated stones, gypsum stuccoes, concrete, wall paintings, archaeological bones and paper. First, the studies aimed at identifying the best treatment conditions (e.g., nature and concentration of the phosphate precursor, solution pH, treatment duration, ionic and organic additions to the phosphate solution, mineralogical composition of the new CaP phases) are summarized. Then, the treatment performance on marble and limestone is reviewed, in terms of protective and consolidating effectiveness, compatibility (aesthetic, microstructural and physical) and durability. Some pilot applications in real case studies are also reported. Recent research aimed at extending the phosphate treatment to other substrates is then illustrated. Finally, the strengths of the phosphate treatment are summarized, in comparison with alternative products, and some aspects needing future research are outlined.

## 1. Introduction

**Figure 1:**
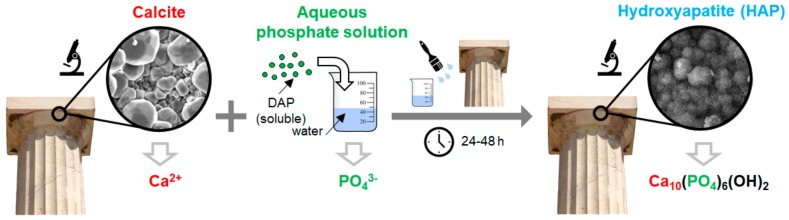
Scheme illustrating *in situ* formation of hydroxyapatite (HAP), as the reaction product between the substrate and an aqueous solution of a phosphate salt (typically DAP).

The use of hydroxyapatite (HAP) and calcium phosphates (CaP) was initially proposed in 2011 for the conservation of carbonate stones (i.e., marble and limestone) [1,2,3,4] and later explored for the conservation of additional substrates, including sandstone [5,6], sulphated stones [7,8,9,10], gypsum stuccoes [10,11], concrete [12], wall paintings [13], archaeological bones [14,15] and paper [16]. With the exception of a few cases where already-formed HAP nanoparticles were used [16], in all the other cases HAP was formed *in situ* from the reaction between the substrate and an aqueous phosphate solution, as schematically illustrated in Figure 1.

In the case of carbonate stones, the need for a new class of conservation treatments was motivated by the lack of fully satisfying solutions for preventing and arresting the weathering processes typically affecting carbonate stones. Marble is mainly affected by dissolution in rain (owing to the relatively high solubility of calcite in water) [17] and by thermally induced micro-cracking (owing to the anisotropic thermal deformation of calcite crystals upon temperature variations) [18]. Limestone is mainly affected by ice and salt crystallization in pores, causing stress [19].

To prevent stone dissolution in rain, protectives are usually applied, with the aim of making the stone surface hydrophobic or forming a surface layer with reduced solubility. To restore cohesion among grains and improve mechanical properties, consolidants are usually applied. Unfortunately, the commercial protectives and consolidants are not fully satisfactory when applied to carbonate stones. In fact, organic products (e.g., acrylic resins) generally lack compatibility with the substrate and durability [20], whereas inorganic treatments (e.g., lime-based, silicate and ammonium oxalate) generally exhibit limited effectiveness on carbonate stones [21].

To overcome the limitations of the available products, the use of CaP for consolidation and protection of carbonate stones was proposed [1]. The idea of using calcium phosphates (ideally HAP, which is the most stable phase at pH > 4 [22]) was conceived as a possible way to improve the ammonium oxalate (AmOx) treatment proposed by Matteini in the 90s [23]. This latter treatment is aimed at artificially forming a layer of calcium oxalate monohydrate (whewellite, CaC_2_O_4_·H_2_O), by reacting the carbonate substrate with an aqueous solution of AmOx [23]. However, the protecting ability of so-formed calcium oxalate layers was found to be lower than expected [24]. This was ascribed to the following reasons [1]: (i) the relatively high solubility of calcium oxalate in water (similar to that of calcite, Table 1); (ii) some mismatch in the lattice parameters of calcium oxalate and calcite (Table 1), which is thought to induce stress at the interface and hence limit the performance of the whewellite layer. Starting from these considerations, HAP (Ca_5_(PO_4_)_3_(OH), usually written as Ca_10_(PO_4_)_6_(OH)_2_ to denote that the crystal unit cell comprises two formula units) was taken into consideration as a potentially better protecting mineral than calcium oxalate [1]. This derives from two considerations: (i) HAP has solubility and dissolution rate much lower than those of calcite (Table 1); (ii) HAP has a good match in lattice parameters with calcite (Table 1), so that formation of a coherent (possibly epitaxial) layer of HAP over calcite can be expected. Research on the possible routes to form HAP starting from calcite was hence undertaken.

Since HAP is the main constituent of human teeth and bones, abundant literature is available about HAP synthesis methods. However, in the case of cultural heritage conservation, strict constraints exist on the conditions that may be employed in the field to form HAP (in terms of temperature, pressure, pH, toxicity, etc.). The present review focuses on this latter topic, whereas methods for HAP synthesis and HAP performance for biomedical applications have been addressed in several previous reviews [22,32].

In the following paragraphs, the studies aimed at identifying the best treatment parameters (e.g., nature and concentration of the phosphate precursor, solution pH, treatment duration, ionic and organic additions to the phosphate solution, mineralogical composition of the new CaP phases) are first summarized. Then, the studies aimed at evaluating the performance of the phosphate treatment for the conservation of marble (i.e., protection, consolidation, arrest and prevention of bowing, functionalization to achieve self-cleaning and antibiotic ability) and for the conservation of porous limestone (i.e., consolidation and ability as a coupling agent for silicate consolidants) are described. Finally, the most recent research aimed at extending the use of the phosphate treatment to substrates other than carbonate stones (e.g., sandstone, sulphated stones, gypsum stuccoes, concrete, archaeologic wall paintings, archaeological bones, paper) are presented. In the conclusions, the strengths of the phosphate treatments are summarized and some aspects needing future research are finally outlined.

## 2. Definition of the Treatment Parameters

### 2.1. Nature of the Phosphate Precursor

Apart from some “biomimetic” (cf. Section 2.8) and “biogenetic” (cf. Section 5.3) routes, all the other methods for *in situ* HAP synthesis involved reaction of the calcium-rich substrate with an aqueous solution of a phosphate salt, providing the PO_4_^3−^ ions necessary to form HAP (Figure 1). Ammonium phosphate salts were selected as the most suitable precursors, as they are highly soluble in water and do not leave undesired cations in the stone, thanks to the volatility of the ammonia and ammonium carbonate originating from the salt dissociation [13].

Among ammonium phosphate salts, diammonium hydrogen phosphate (DAP, (NH_4_)_2_HPO_4_)) was the first to be investigated [1,3]. Based on biomedical literature, HAP can be formed by reacting calcite with an aqueous solution of DAP according to the reaction [33]:

10CaCO_3_ + 5(NH_4_)_2_HPO_4_ → Ca_10_(PO_4_,CO_3_)_6_(OH,CO_3_)_2_ + 5(NH_4_)_2_CO_3_ + 3CO_2_ + 2H_2_O. (1)

The by-products of this reaction (ammonium carbonate and carbon dioxide, alongside water) are all innocuous and volatile, so that no undesired residue is expected to remain in the stone. The reaction, originally proposed at 40 °C [33], was found to occur also at room temperature [1]. The so-formed HAP is typically non-stoichiometric, as CO_3_^2−^ ions from CO_2_ in the atmosphere can partially replace PO_4_^3−^ and OH^−^ ions, thus leading to formation of carbonated HAP. Since carbonated HAP has a higher solubility than stoichiometric HAP [34], its formation would negatively affect the treatment performance, hence it should be prevented (cf. Section 2.9). As discussed in detail in Section 2.9, alongside HAP several metastable CaP phases may form from reaction 1, depending on the reaction conditions (e.g., pH, time, degree of supersaturation) [1]. Such metastable phases are expected to finally convert into HAP, the most stable CaP phase, via dissolution and reprecipitation processes [1]. However, because some of these metastable phases have high solubility in water (Table 2), direct formation of stable, insoluble phases should be promoted [1,35].

As an alternative to DAP, ammonium dihydrogen phosphate (ADP, NH_4_H_2_PO_4_) [3,34] and triammonium phosphate (TAP, (NH_4_)_3_PO_4_) [4,5,34] have also been investigated as possible precursors. The main reason for testing a different precursor was to favourably modify the ratio between PO_4_^3−^, HPO_4_^2−^ and H_2_PO_4_^−^ ions formed when the precursor dissociates in water. In fact, in a 1 M DAP solution (without any pH adjustment), only ~0.3% of phosphate ions are in the form of PO_4_^3−^ (needed to form HAP), while the majority is in the form of HPO_4_^2−^ (~99%) and H_2_PO_4_^−^ (~0.7%) [34]. Because only PO_4_^3−^ ions are needed to form HAP, TAP was expected to provide the best results. However, the relative amount of PO_4_^3−^, HPO_4_^2−^ and H_2_PO_4_^−^ ions actually depends on the solution pH [22,32], hence the nature of the phosphate precursor is basically irrelevant as long as pH is controlled [34]. Consequently, experiments on limestone consolidation by treatment with solutions of DAP at pH 8 or ADP at pH adjusted to 7–8 gave similar results, in terms of phase composition and increase in mechanical properties [3].

### 2.2. pH of the Phosphate Solution

Without any pH adjustment, a 1 M DAP solution has pH 8.3. As mentioned in the previous paragraph, in these conditions only 0.003 M PO_4_^3−^ ions are created from DAP speciation [34]. In this pH range, formation of HAP but also other CaP phases is expected [32]. At a higher pH, deprotonation of HPO_4_^2−^ is favoured and so should be the formation of HAP [3,32,34]. Therefore, the effect of pushing pH towards more basic values was investigated.

When the pH of a 0.1 M DAP solution (also containing 0.1 mM CaCl_2_ as a calcium source, cf. Section 2.5) was increased from 8 to 11 using NaOH, complete and uniform coverage of a marble surface was obtained by a CaP phase different from that formed at pH 8 [37]. This new phase was identified by FT-IR as nanocrystalline or disordered HAP but also other minor phases were likely present, as suggested by their different morphology and their dissolution after exposure to a slightly acidic solution [37]. Consequently, such pH modification was regarded as not promising [37].

As an alternative to NaOH (introducing Na^+^ ions in the solution), the use of NH_4_OH was investigated, because in this case no influence from the cation is expected [38]. Increase of pH from 8 to 10 was investigated for a 0.1 M DAP + 0.1 mM CaCl_2_ solution, with and without addition of ethanol to favour CaP formation (cf. Section 2.7) [38]. Whereas no CaP phases were formed at pH 8, abundant new phases were formed at pH 10 but in all cases the excessive growth of the coating caused diffused cracking [38]. To prevent cracking, diminishing the DAP concentration was attempted (always with addition of CaCl_2_ in 1:1000 ratio to DAP and with 6 vol % ethanol to favour CaP formation): diminishing the DAP concentration by 10 times (0.01 M DAP) again led to cracking of the coating, whereas diminishing by 100 times (0.001 M DAP) led to no coating formation, because of the insufficient amount of PO_4_^3−^ present in the solution [38]. Because the promotion of CaP formation was always linked with cracking (detrimental to achieve high resistance to dissolution in rain), none of these attempts to increase the pH was regarded as promising.

### 2.3. Concentration of the Phosphate Solution

A wide range of DAP concentrations has been explored, ranging from 0.1 M [34,37,38,39] to 3.7 M [1] (corresponding to the saturation concentration at room temperature).

The use of solutions with low concentration has been pursued to reduce cracking of the CaP coating: the lower the concentration, the thinner the coating and, hence, the lower the tendency to crack [40]. However, the need to have a sufficient amount of PO_4_^3−^ ions (produced by DAP dissociation only in very low amounts, if pH is not adjusted [34]) has pushed the use of DAP solutions with higher concentrations. The effects of using progressively increasing DAP concentrations are summarized in the following.

When a 0.1 M DAP solution was used to react marble, no complete coverage of the surface was obtained after 24 h [34]. An improvement in the surface coverage was achieved by adding 20 mM CaCl_2_ to the 0.1 M DAP solution but in this case HAP and very soluble mono-calcium phosphate mono-hydrate were formed [2]. Complete surface coverage was obtained by octacalcium phosphate (OCP, Ca_8_H_2_(PO_4_)_6_·5H_2_O, having very low solubility, Table 2), by treating marble with a solution containing 0.1 M DAP + 0.1 mM CaCl_2_ in 10 vol % ethanol [38,39]. This was possible thanks to the ability of ethanol to favour CaP formation [37], because ethanol molecules weaken the hydration shell of phosphate ions in solution [41] (cf. detailed discussion in Section 2.7). As an alternative to ethanol addition, abundant formation of CaP phases was obtained also when the pH of a 0.1 M DAP + 0.1 mM CaCl_2_ solution was increased from 8 to 10 [38] or 11 [37]. However, the coating formed at these pH values was cracked and/or contained soluble phases, hence this route was regarded as not promising.

By increasing the DAP concentration from 0.1 M to 1 M, an increase in the amount of new CaP phases was found [34]. However, because of the increased thickness of the coating, also the film tendency to crack was increased [38]. Coatings formed at even higher DAP concentration (3 M DAP, followed by limewater application [42]) exhibited diffused cracking [43] but still a remarkable consolidating effectiveness. Because the consolidating action derives from the ability of the new CaP phases to seal the tips of cracks among grains (even though a discontinuous coating is formed), a steady increase in mechanical consolidation (assessed by ultrasound) was found for increasing DAP concentration, both in porous limestone [1] and in marble [44].

To prevent cracking occurring in thick films formed at high DAP concentrations, the superimposition of two thinner films (each formed at low DAP concentration) was investigated, with good results [37]. Alternatively, a reduction in cracks and pores was obtained using high DAP concentrations and introducing nanoparticles in the DAP solution [45]. Indeed, nanoparticles are thought to favour HAP nucleation by acting as seeds and to reduce shrinkage during drying [45]. Moreover, by addition of nanoparticles new functionalities (e.g., self-cleaning ability) can be obtained, as described in detail in Section 3.4.

### 2.4. Duration of the Reaction

Reaction times ranging from 1 h [34] to 14 days [2] have been investigated.

Even though reaction times of 3 h [46] and 8 h [3] have been proposed as sufficient to achieve suitable limestone consolidation, nonetheless treatment for at least 24 h is generally recommended [1,34,38,42,43]. Indeed, after treatment for 24 h, marble surface was almost completely covered by new CaP using a 1 M DAP + 1 mM CaCl_2_ solution [34] and completely covered using a 0.1 M DAP + 0.1 mM CaCl_2_ in 10 vol % ethanol solution [38]. In terms of improvement in mechanical properties, about 80% of the increase achieved after 8 days of reaction with a 1 M DAP solution was already registered after 24 h and 90% after 48 h [1]. A treatment duration of 24–48 h is also feasible in the onsite practice of monument conservation.

Passing from 24 to 48 h, a change in morphology of the new CaP phases formed by reacting calcitic powders with a 10% (0.76 M) DAP solution has been reported in Reference [35]. After 24 h of reaction (with no stirring), the calcitic particles were covered with a shell made of brushite and OCP, over which a layer of HAP aggregates with plate-like appearance developed. After 48 h, the morphology of the HAP layer changed into needle-like individuals [35].

Some evolution in phase formation at longer periods has also been reported [9,46]. In the case of a calcarenite covered with a gypsum crust, treated with a 3 M DAP solution for 60 min, phase evolution (assessed by X-ray diffraction, XRD) continued after the end of the treatment application and apparently was completed after 1 week [9]. In the case of a marlstone treated with a 1 M DAP solution, after 1 year HAP formation (assessed by Fourier-transform infrared spectroscopy, FT-IR, and scanning electronic microscopy with energy dispersive spectrometry, SEM-EDS) was found to be more evident than right after the treatment, which was attributed to the likely presence of unreacted DAP remaining in the stone (however, below the FT-IR and XRD detection limit) [46]. Unfortunately, no data were reported on the possible improvement in the quality of the consolidation, following additional CaP formation at long periods.

### 2.5. Calcium Ion Addition

According to reaction 1, calcium ions necessary to form HAP only derive from dissolution of the substrate. However, if calcium ions are externally provided in the DAP solution, two advantages can be achieved: (i) no dissolution of the substrate takes place, which is positive because some concerns may arise when carved surfaces are treated (however, even if no calcium source is added, only millimolar quantities of calcite need to dissolve for the HAP-forming reaction) [2]; (ii) a faster and more complete coverage of marble surface can be achieved [2,34].

Several sources of calcium ions have been investigated, namely CaCl_2_ [2,34], Ca(NO_3_)_2_ [2], Ca(OH)_2_ [47], calcium gluconate [34] and calcium formate [34]. The investigated concentrations of these calcium sources ranged from 0.5 to 2 mM for a DAP concentration of 0.5 to 1 M (in all cases, the rationale was to introduce as many calcium ions as possible, without causing precipitation directly in the beaker) [2,34,47]. The best results were obtained when 1 mM CaCl_2_ was added to a 1 M DAP solution. In these conditions, the rate of nucleation and the density of the new CaP film was found to increase significantly [2,34].

An important consequence of the addition of CaCl_2_ to the DAP solution is a change in the composition of the CaP phases formed after treatment. Whereas only HAP was found when marble was treated with a solution of 1 M DAP, both HAP and OCP were found when 1 mM CaCl_2_ was added to the 1 M DAP solution [34]. This was ascribed to the fact that the increase in solution supersaturation evidently permits less stable, more soluble phases to form [34]. As reported in Table 2, even though OCP has a higher solubility product than HAP, it is still much less soluble than calcite. Therefore, OCP formation is not expected to negatively affect the coating performance, especially because it provides more uniform coverage of the marble surface [34].

To purposely introduce chlorides into stone might sound risky for its conservation. However, no traces of chloride salts have been found by XRD [34]. In some cases (but not systematically [48]), a small chloride peak was found by EDS in samples treated with 1 M DAP + 1 mM CaCl_2_ [34]. Because these chloride traces were found even after extensive washing, it is believed that chlorides are incorporated into the HAP crystal, which can undergo ionic substitutions [48]. When stone was treated with lower CaCl_2_ concentrations (e.g., 0.1 M DAP + 0.1 mM CaCl_2_ in 10 vol % ethanol [38]) no chloride traces were found. Therefore, no risk coming from chloride salts seems likely, because the millimolar chloride concentrations added to the DAP solution are either washed away by rinsing at the end of the treatment or incorporated into the HAP crystal [48].

As an alternative to the addition of calcium sources to the DAP solution, the possible pre-treatment of the stone with limewater (i.e., a saturated solution of Ca(OH)_2_) [47] or nanolimes (i.e., a dispersion of nanoCa(OH)_2_) [4,5] has been investigated as well. In both cases good results were obtained but no substantial advantage with respect to the addition of a calcium sources directly into the DAP solution has been highlighted.

Application of a limewater poultice after treatment with a 3 M DAP solution has been investigated as well [42,43,44,45,49,50,51]. In this case, the limewater poultice, applied after treatment with the DAP solution and drying, has the twofold purpose of (i) providing additional calcium ions for the reaction with unreacted DAP and (ii) removing all the unreacted DAP during drying, as the poultice is left to dry in contact with the stone [42]. This method was found to be successful in boosting HAP formation and removing unreacted DAP, so that no soluble phosphates were detected in the stone at the end of the treatment, even when a very high DAP concentration is used [51].

### 2.6. Other Ionic Additions

To inhibit dissolution of the calcitic substrate, the addition of CO_3_^2−^ ions was explored [34]. The addition of 15–150 μM (NH_4_)_2_CO_3_ to a 1 M DAP + 1 mM CaCl_2_ solution was investigated [34]. Compared to the same formulation with no carbonate addition, quicker formation of the CaP layer was observed but also reduced protective ability, likely because of increased film porosity and cracking during drying [52]. A further aspect discouraging carbonate addition to the DAP solution is that carbonate ions might be incorporated into the HAP lattice, leading to formation of carbonated HAP, more soluble than stoichiometric HAP [34]. However, the addition of ammonium carbonate was found to be unnecessary, because, based on theoretical calculations, when 1 mM CaCl_2_ is added to a 1 M DAP solution, the substrate dissolution is already suppressed [34].

To improve the lattice match between HAP and calcite and hence to favour heterogeneous nucleation of HAP over calcite, the addition of Sr^2+^ [34,37], Mg^2+^ [34,37] and Al^3+^ [37] ions, both at pH 8 and pH 11, was explored. The effects of adding 0.1–1 mM SrCl_2_ or MgCl_2_ to a 1 M DAP solution (with and without 1 mM CaCl_2_ addition) were investigated in Reference [34]. Strontium was incorporated into the HAP crystal, while magnesium was not; however, in both cases a lower reaction rate was found (i.e., less complete surface coverage was observed after 24 h) [34]. Correspondingly, no improvement in the ability to protect marble from dissolution was registered [52]. Similarly, no benefit was achieved when 0.010–0.025 mM AlNO_3_ were added to a 0.1 M DAP also containing 0.1 Mm CaCl_2_, both at pH 8 or 11 [37].

Encouraging results in terms of coating formation and mechanical consolidation were obtained when marble was treated with a 0.1 M DAP + 0.1 mM Al(NO_3_)_3_∙9H_2_O solution in 10 vol % ethanol [53]. In this case, the aluminium source was added instead of (and not in addition to) the calcium source, with the aim of forming aluminium phosphates (ideally, berlinite, AlPO_4_) and not calcium phosphates. This choice derived from the fact that berlinite shows a very good match in lattice parameters with calcite, even better than HAP [53]. In fact, HAP exhibits a 5% lattice mismatch with calcite, which might be large enough to result in stress if the layer grows more than a few nm thick. Berlinite only shows a 1% mismatch with calcite and a solubility product ~10^−22^, hence much lower than that of calcite (5 × 10^−9^) [53]. Further research is needed to confirm the promising results obtained in the cited study [53] about aluminium phosphates.

As described in detail in Section 2.8, incorporation of fluorine ions in the apatite crystal was investigated in Reference [54], with the aim of forming fluorapatite (instead of HAP).

### 2.7. Organic Additions

The addition of alcohols to DAP solutions has proven to have a strong effect on the nucleation and the microstructure of the new CaP phases [37,38]. The addition of even small amounts of ethanol (0.1 up to 20 wt %) to a 0.1 M DAP + 0.1 mM CaCl_2_ solution allowed complete coverage of calcitic powders [37]. Notably, no coating over a marble surface was obtained with the same DAP and CaCl_2_ concentrations when no ethanol was added [38,39]. In the case of massive marble, adding 10 vol % ethanol to a 0.1 M DAP + 0.1 mM CaCl_2_ solution caused formation of an OCP coating with much denser microstructure, compared to the HAP + OCP coating formed by reaction with a 1 M DAP + 1 mM CaCl_2_ solution [38]. The positive effect of ethanol on the coating microstructure is thought to derive from the weakening effect of ethanol molecules on the hydration sphere of phosphate ions in solution [41], with the result that formation of CaP is favoured. However, ethanol is also adsorbed on the surface of calcite [55], which might be counterproductive towards obtaining a non-porous, well adherent coating over marble. Because isopropanol has lower adsorption affinity to calcite than ethanol (i.e., isopropanol binds less strongly than ethanol) [56], using isopropanol instead of ethanol was attempted. By adding 10 vol % isopropanol to a 0.1 M DAP + 0.1 mM CaCl_2_ solution, an even denser OCP coating was obtained [38]. A significant reduction in the coating porosity is important towards obtaining effective protection of marble from dissolution in rain, because the presence of porosity might allow acid to permeate through the coating and trigger dissolution of the substrate [38]. In spite of the higher solubility of OCP compared to calcite, non-porous OCP coatings were found to provide greater acid protection than porous HAP + OCP coatings [38].

The addition of 0–0.2 M cetyltrimethylammonium bromide (CTAB) to a 1 M DAP solution was investigated in Reference [57]. CTAB molecules are composed of two parts (a positively charged hydrophilic head and a hydrophobic tail), that form micelles through a self-assembly process [57]. The surface of these micelles attracts HPO_4_^2−^ ions originating from DAP dissociation in water [57]. The hypothesis of this study was that CTA^+^-HPO_4_^2−^ micelles act as nucleation centres, so that dissolved Ca^2+^ from the substrate immediately combine with HPO_4_^2−^ ions [57]. However, since HAP requires PO_4_^3−^ ions and not HPO_4_^2−^ ions, it is not exactly clear how this procedure promotes formation of HAP. It is noteworthy that in the cited study HAP was not identified by XRD, either because the amount was too low or because HAP was poorly crystalline [57]. The addition of 0.1 M CTAB to a 1 M DAP solution led to a significant reduction in specific surface area and pore volume, linked to an increase in the size of the crystals [57]. The HAP layer formed with the addition of CTAB to the DAP solution slightly increased the acid resistance and the mechanical consolidation of the treated limestone powder, compared to the use of DAP alone [57].

### 2.8. Biomimetic Routes

Some “biomimetic” or “metasomatic” methods to form HAP over marble surface have been explored as well. The use of a solution of collagen (C_12_H_18_O_4_N_3_) followed by application of a 10 mM CaCl_2_ + 6 mM DAP solution (the Ca/P ratio corresponding to that of HAP) was investigated, with the expectation that HAP would grow within the collagen network [58]. The resulting HAP coating was reportedly porous and net-like [58].

Following a similar approach, in another study [54] fluorapatite (Ca_10_(PO_4_)_6_F_2_) was formed by reacting marble with a solution containing 0.1 g/L of Type 1 collagen + 5 g/L of ATP + 0.6 g/L of NH_4_F [54]. The resulting layer of fluorapatite (about ~60 μm thick) was reportedly coral-like, without any crack or detachment [54]. The role of collagen was found to be essential because, when the same concentrations of TAP and NH_4_F were used to treat marble in the absence of collagen, a cracked and partly detached fluorapatite layer was obtained [54]. The role of collagen is to promote the direct formation of fluorapatite on the marble surface, by acting as a template. According to the mechanism proposed in the cited paper, in the presence of collagen calcium ions dissolved from the substrate adsorb at active sites (such as carboxyl groups) on the matrix and react with phosphate and fluoride ions, thus leading to fluorapatite formation [54]. As discussed in Section 3.1, the fluorapatite film is reportedly able to protect marble from dissolution in acid but no direct experimental test was carried out in the Reference [54] to check the actual protective effectiveness. In any case, it should be noted that the real applicability of fluorapatite in practical situations is limited by the high toxicity of ammonium fluoride (NH_4_F), whose health effects are defined as “very hazardous” in the Material Safety Data Sheet (MSDS).

A “metasomatic reaction” between marble and a monocalcium phosphate solution (1 g/L) for 72 h was also explored [59]. The resulting HAP layer, about 100 μm thick, reportedly exhibited no visible crack or peeling but was porous [59]. As discussed in Section 3.1, good protective efficacy was expected from this HAP layer [59] but no direct experimental test was carried out to measure its actual protective efficacy.

A different route was investigated in Reference [12], where formation of “biogenetic” HAP through the action of living organisms was explored for the conservation of cement concrete, as described in detail in Section 5.3.

### 2.9. Nature of the New Calcium Phosphates

The mineralogical composition of the new CaP formed after treatment is a fundamental aspect, which depends on all the above-discussed parameters. The mineralogical composition of the new phases is fundamental, because their solubility in water may vary by several orders of magnitude. Formation of highly soluble CaP might render a protective or consolidating treatment useless or even counterproductive. A list of the different CaP phases and their water solubility is given in Table 2.

The conclusive identification of the new CaP phases is a very challenging task, because these phases are often present in very low amounts, have similar crystal structures and coexist in complex mixtures. Experimental techniques that have been used for their identification include XRD on powders [3,35], grazing incidence XRD on marble surfaces [34,38], FT-IR on powders [37,49,60] and on stone surface [35], μ-Raman on surfaces [43] and on cross-sections [35] and EBSD on fracture surfaces [1]. However, for a conclusive identification, a multi-analytical approach is recommended [35]. A summary of the different CaP phases formed after different treatments and the analytical techniques used for their identification is reported in Table 3.

**Figure 2:**
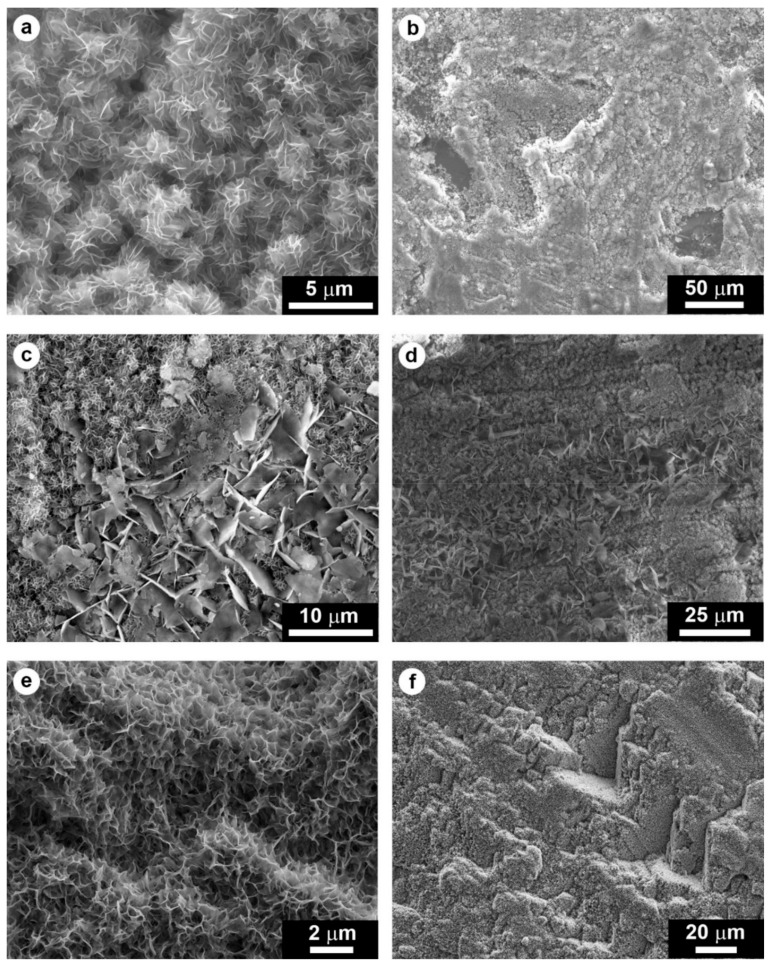
Scanning electron microscopy (SEM) images of CaP coatings with different compositions (determined by XRD): (**a**,**b**) HAP coating formed over Carrara marble after treatment with a 1 M DAP solution for 24 h; (**c**,**d**) HAP + octacalcium phosphate (OCP) coating formed over Carrara marble after treatment with a 1 M DAP + 1 mM CaCl_2_ solution for 9 h (**c**) and 24 h (**d**) - by comparison with the morphology of the HAP coating illustrated in (**a**,**b**), the small flakes in (**c**) were identified as HAP and the large flakes as OCP [34]; (**e**,**f**) OCP coating formed over Carrara marble after treatment with a 0.1 M DAP + 1 mM CaCl_2_ in 10 vol % ethanol solution for 24 h. Images (**a**–**d**) adapted from reference [34] with permission from Elsevier.

Distinction among different CaP phases based on their microscopic morphology is challenging, because of their similarity. In a case where phase identification had been carried out by XRD, the assignment of the different morphologies observed by SEM to the identified phases (HAP and OCP) was attempted based on a process of elimination [34]: as described in Figure 2c, the small flakes were identified as HAP and the large flakes as OCP. Using EDS in combination with SEM has been attempted as well [4] to distinguish among different CaP phases based on their different Ca/P ratio (Table 2). However, depending on the thickness of the CaP coating and on the interaction volume between the electron beam and the sample (from which the characteristic X-rays detected by EDS are emitted), a conclusive identification of the different CaP phases is made difficult by the possible influence of the calcium-rich substrate on the measured Ca/P ratio.

HAP, being the most stable phase in aqueous solutions at pH > 4 [22], is the most desirable phase to form. However, other CaP, more soluble than HAP but still less soluble than calcite, are not undesirable, especially if their formation allows more complete coverage of the marble surface and/or reduced coating porosity. This is the case of OCP, whose formation is favoured by CaCl_2_ addition (leading to improved coverage of marble surfaces) [34] and by ethanol addition (leading to improved density of the coating) [38]. On the contrary, formation of phases with solubility higher than the substrate should be prevented, as they are expected to dissolve easily upon exposure to rain. For instance, when limestone was treated with a solution of ADP at pH 5.6–6, brushite was formed alongside HAP [3], which is expected to lead to reduced durability of the treatment.

Formation of brushite even in a pH range and for a Ca/P ratio that should favour HAP has been reported [10]. This has been ascribed to the fact that DAP dissociation in water leads to formation of a huge amount of HPO_4_^2−^ ions and a minor amount of PO_4_^3−^ ions: since precipitation of brushite requires HPO_4_^2−^ ions and HAP requires PO_4_^3−^ ions, the former mineral is favoured [10]. In this way, even if HAP is thermodynamically the most stable phase, brushite is kinetically favoured [9]. A further possible reason for precipitation of brushite and other acid CaP (e.g., OCP and ACP) has been identified in the progressive release of H^+^ from HPO_4_^2−^ ions [35]. These H^+^ ions are responsible for local acidification of the DAP solution at the interface with the stone, which may lead to local pH values unsuitable for HAP formation [35]. An acidic micro-environment is also responsible for creation of corrosion marks on the surface of the treated stone [35].

Among other phases formed after treatment with DAP solutions, β-TCP has also been reported [9,43]. However, because β-TCP cannot be precipitated via wet synthesis at room temperature but can only be formed at high temperature [22], its formation after treatment with DAP solutions is unlikely. This confirms the difficulty of correctly identifying the new CaP phases formed after treatment and the need of adopting a multi-analytical approach to achieve a reliable identification [35].

A mechanism of CaP phase formation and evolution has been proposed in Reference [35]. In the case of calcitic powders reacted with a 10% (0.76 M) DAP solution for 24, 48 and 168 h [35], brushite and OCP (being more acidic) are the first phases to form, directly at the calcite-solution interface [35]. Brushite and OCP form a compact shell around calcite particles (thickness ~1 μm after 24 h and 2–3 μm after 48 h). Over this inner shell, HAP nucleation and growth progressively take place, leading to formation of a thicker, less compact outer shell [35]. This HAP layer is formed by plate-like aggregates after 24 h and needle-like individuals after 48 h [35]. Alongside HAP, also ACP was detected [35]. Notably, brushite was found to have transformed into HAP after 6 months [35]. However, it should be noted that the nature of the CaP phases and the proposed mechanism of phase evolution are strongly dependent on the specific treatment conditions, especially the absence of any external calcium source and the absence of stirring during the reaction [35].

Another important issue is the possible permanence of unreacted DAP inside the stone at the end of the treatment. In fact, being reach in phosphorus and nitrogen, unreacted DAP may favour biological growth [62]. In the case of a marlstone rock-cut chamber tomb in Cyprus, 1 year after consolidation with a 1 M DAP solution, some traces of biological growth were found in some treated areas but not all [46]. DAP residues have been detected after reacting powders with a 0.76 M DAP solution [35] and marble samples with a 1 M DAP + 1 mM CaCl_2_ solution [34] but in both cases the DAP residues were removed by rinsing with water at the end of the treatment [34,35]. In the case of more concentrated DAP solutions (e.g., 3 M), the application of a limewater poultice at the end of the treatment proved to be effective in removing unreacted DAP, thanks to DAP dissolution in limewater and limewater migration into the poultice during drying [43,51]. Nonetheless, a study was specifically carried out to identify the most effective way to prevent biological growth linked to the possible presence of unreacted DAP inside treated stone [62], as described in detail in Section 3.7.3.

## 3. Marble

The phosphate treatment has been investigated on marble with the aim of providing several functions: (i) protection from dissolution in rain (Section 3.1); (ii) mechanical consolidation (Section 3.2); (iii) arrest and prevention of bowing (Section 3.3); (iv) functionalization to provide self-cleaning ability (Section 3.4) and anti-fungal ability (Section 3.5); (v) desulphation (discussed in detail in Section 5.3). For any of these purposes, the phosphate treatment must ensure suitable compatibility (Section 3.6) and durability (Section 3.7).

### 3.1. Protection from Corrosion

**Figure 3:**
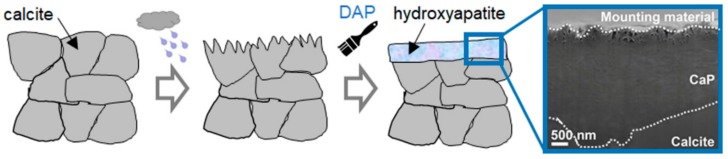
Scheme illustrating the protective action of the phosphate treatment, with an example of a focused ion beam (FIB) SEM cross section of the protective layer formed over marble.

The protective action of the phosphate treatment is schematically illustrated in Figure 3. To achieve a satisfactory protective action, the coating formed over the marble surface needs to meet several requirements: (i) the new CaP phase needs to be less soluble than calcite; (ii) the coating needs to be continuous (i.e., there should be no bare areas, from where the attack on the substrate may start); (iii) the coating needs to be crack-free and pore-free (because cracks and pores may allow rain to reach the substrate and trigger dissolution).

A reduction of ~40% in the rate of acid attack was found for marble treated with a 1 M DAP + 1 mM CaCl_2_ solution [52]. The coating formed in these conditions is composed of HAP and OCP [34], both far less soluble than calcite (Table 2). However, the protective ability of this coating was lower than expected based on the solubility product of HAP and OCP, compared to calcite. The reduced performance was attributed to the presence of some residual uncoated areas [52] and to the presence of cracks (likely originating during drying) and pores [38].

To prevent cracking, the use of lower DAP concentrations (leading to formation of a thinner coating, less prone to cracking) was envisaged [37]. However, using a 0.1 M DAP + 0.1 mM CaCl_2_ solution led to formation of a patchy coating, with reduced protective ability [37]. Significantly better protection was obtained with the addition of a small amount of ethanol to the DAP solution, thanks to the beneficial effect of ethanol on CaP formation (cf. Section 2.7). Double application of a 0.1 M DAP + 0.1 mM CaCl_2_ solution, also containing 0.5 wt % ethanol during the first application (and not during the second one, to avoid excessive film growth), was found to provide significant protection, both on calcitic powders [37] and massive marble samples [63].

**Figure 4:**
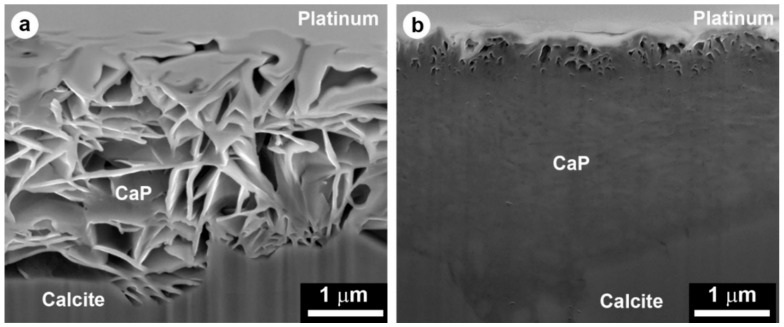
FIB-SEM cross sections of coatings formed by reacting marble for 24 h with solutions containing (**a**) 1 M DAP + 1 mM CaCl_2_ and (**b**) 0.1 M DAP + 0.1 mM CaCl_2_ in 10 vol % ethanol. The much denser coating in (**b**) was formed thanks to the beneficial effect of ethanol, as described in detail in Section 2.7. Images adapted from reference [38] with permission.

Treatment with a 0.1 M DAP + 0.1 mM CaCl_2_ solution in 10 vol % ethanol provided greater protection than treatment with 1 M DAP + 1 mM CaCl_2_ solution without alcohol [38]. This was possible as the former treatment led to formation of an OCP coating that was crack-free (thanks to the reduction in DAP concentration) and pore-free (thanks to the beneficial effect of ethanol) [38]. The different microstructures of the coatings formed in the two cases are illustrated in Figure 4.

Even with the addition of ethanol, some calcite grains were found to remain uncoated after treatment, which was ascribed to their specific crystallographic orientation. Whereas epitaxial growth might occur over grains with favourable orientation (e.g., when the distance between carbonate groups in the calcite crystal closely matches the distance of phosphate groups in the CaP crystal) and the growing CaP coating can then spread to the adjacent grains with random orientation, when the coating reaches calcite grains with unfavourable orientation (i.e., atomic distances very different from those of the CaP crystals) its further development might be impeded [38].

In the above-mentioned studies, the protective action of the treatments was investigated by immersing samples in a beaker containing a slightly acidic solution simulating rain (at pH ~5) and measuring the pH increase over time [37,38,52]. As Ca^2+^ ions are released from calcite, H^+^ ions are consumed and the solution pH increases [52]. Even though this method is very useful to screen among different formulations of the protective treatments, it has the limitation that dissolved Ca^2+^ ions are retained in the solution, which affects the reaction kinetics. In fact, they reduce the diffusion rate of products away from the calcite-solution interface, thus reducing the dissolution rate [52]. Consequently, testing the coating protective effectiveness in more realistic conditions is recommended.

In a recent study, a custom-designed apparatus, able to drip a solution at fixed pH and fixed rate over numerous samples at a time, was used to evaluate the protecting ability of different formulations of the phosphate treatment [63]. This apparatus has the advantage that the solution dripped onto the samples is always at the same fixed pH and contains no Ca^2+^ ions (unlike the tests carried out in beakers, where pH progressively increases and Ca^2+^ ions progressively accumulate, which decreases the solution aggressiveness). Moreover, the rain-simulating apparatus allows the runoff solution to be collected for analysis of the leached ions (e.g., Ca^2+^ ions), which can be used to quantify the protective ability [63]. By treatment with a 0.1 M DAP + 0.1 mM CaCl_2_ solution applied twice, the first time also with 0.5 wt % ethanol, a reduction of ~40% in the amount of dissolved Ca^2+^ ions was obtained, compared to untreated marble [63]. This formulation performed better than treatment with a 3 M DAP solution, followed by limewater application, because in this latter case the HAP coating was cracked [63].

Notably, both in the simplified acid attack tests performed by immersing samples in an acidic solution in a beaker [37,38] and in the more realistic tests performed using the rain-simulating apparatus [63], the CaP coatings formed by the phosphate treatment provided greater protection than the AmOx treatment, which is currently the most commonly used inorganic treatment for marble protection. The limited protection of the newly formed calcium oxalate layer is thought to be a consequence of its relatively high solubility, similar to that of calcite (Table 1) [38].

In some studies, it was reported that marble covered with a layer of HAP [59] or fluorapatite [54] is able to resist acidic solutions with pH as low as 4.2 [59] or 4 [54], respectively, whereas untreated marble is corroded at pH 6.5 [59] or 5.5 [54]. Similarly, in Reference [58] it was estimated that marble covered with a layer of HAP (formed by treatment with a 10 mM CaCl_2_ + 6 mM DAP solution) can resist H_2_SO_4_ at pH 0.8, whereas untreated marble is corroded in H_2_SO_4_ at pH 2.5. However, all these estimations of the protective ability of a HAP/fluorapatite coating were based on theoretical considerations and in no case were experimental tests of acid resistance carried out [54,58,59]. Considering that HAP formed by the above-mentioned methods was reportedly porous and net-like [58,59] and fluorapatite was coral-like [54], the actual protective ability of these films is expected to be lower than theoretical considerations would suggest, which makes specific experimental tests indispensable.

#### Combination of Phosphate and Oxalate Treatments

With the aim of improving the protective ability of the phosphate treatment [37] and achieving both protection and consolidation [61], sequential application [37] and simultaneous application [37,61] of the phosphate treatment and the oxalate treatment have been explored.

In Reference [37], sequential application of AmOx and DAP was tested on calcitic powders, in an attempt to combine the ability of whewellite to uniformly cover the marble surface, with the much lower solubility of HAP (which, in turn, leaves some calcite grains uncoated, depending on their crystallographic orientation [38]). The sequential treatment consisted in first applying a 5 wt % AmOx solution for 24 h, then (after drying) applying a solution containing 0.1 M DAP + 0.1 mM CaCl_2_ + 0.5 wt % ethanol for 24 h [37]. However, the HAP coating on top of the whewellite coating was patchy and in some areas the whewellite coating itself spalled off because of the application of the DAP solution, so that some bare areas could be observed (unlike the case of the oxalate treatment alone) [37]. As a result, the protective ability was lower than that of the HAP-treatment alone [37]. In fact, the dissolution of the whewellite coating, initiated where some defect in the HAP coating was present, likely caused the detachment of the HAP layer, thus favouring dissolution of the substrate [63].

The same sequence (first, the oxalate treatment and, second, the phosphate treatment) was tested in Reference [61] on tablets of compacted calcite powder and naturally decayed Carrara marble. After application of a 5% *w*/*v* AmOx solution, followed (after drying) by a 5% *w*/*v* DAP solution, only whewellite was found down to a depth of 1 mm, with no CaP formation [61]. This was ascribed to that fact that AmOx reacts quickly with calcite to form a layer of whewellite, which reduces the stone porosity [61]. As a result, when the DAP solution is applied, no more calcium ions are available on the surface to form CaP and penetration of the DAP solution deeply into the substrate is impeded, because the pores are occluded by the newly formed whewellite [61]. Similarly, when the two treatments were applied simultaneously (solution containing 5% *w*/*v* AmOx and 4% *w*/*v* DAP), only whewellite and no CaP was detected down to a depth of 0.9 mm, because AmOx reacts more quickly than DAP [61]. When the two treatments were applied in the reversed order (i.e., first a 5% *w*/*v* DAP solution, then a 5% *w*/*v* AmOx solution), with the aim of first consolidating the substrate by the DAP treatment and then providing protection by the AmOx treatment, HAP was found down to a depth of 2.5 mm and whewellite down to 1.3 mm [61]. This was ascribed to the DAP solution being able to penetrate deeply into the substrate, because of its reduced reaction speed, while whewellite formation was made possible by the subsequent reaction between calcium ions not previously involved in HAP formation and oxalate ions in the AmOx solution [61].

### 3.2. Consolidation

**Figure 5:**
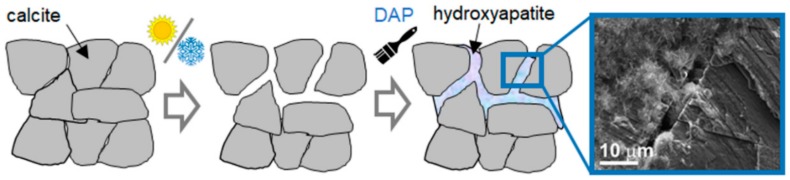
Scheme illustrating the consolidating action of the phosphate treatment, with an example of a SEM image showing HAP growth inside a crack in weathered marble.

The consolidating action of the phosphate treatment is schematically illustrated in Figure 5. The ability of the phosphate treatment to increase cohesion among calcite grains, thus improving mechanical properties, has been measured on samples that resemble naturally weathered marble in a more or less realistic way. A summary of the consolidating effectiveness of the phosphate treatment on different substrates, as well as the depth of penetration of the DAP solution and/or the formation of new CaP, is reported in Table 4.

In the case of compacted powders imitating loose calcite grains in weathered marble, a good consolidating ability was reported for a two-step treatment, consisting in preliminary soaking with nanolimes and subsequent application of a 5% TAP solution [4]. The resistance to material loss by scotch tape test, the compressive strength and the resistance to freeze-thaw cycles were improved after treatment (in particular, the compressive strength reached 4.5 MPa) [4]. It should be noted that, initially, the compacted powders had basically no cohesion.

To achieve improved similarity to real decayed marble, two types of artificially weathered samples have been used: uniformly weathered samples [43,44,60] and samples with a gradient in properties [38,65]. In either case, the aim of consolidation is to bring mechanical properties back to the condition before weathering [49], without causing undesired over-consolidation (strengthening is considered excessive when increases in mechanical properties exceed 125–150% of the initial value [66]).

Uniformly weathered samples were produced by heating in an oven at 200–400 °C, to induce micro-cracking that resembles that of naturally weathered marble [43,44,60]. This artificial weathering method is based on the anisotropic deformation of calcite crystals upon heating [18], resulting in stress at grain boundaries and consequent formation of new micro-cracks [67]. The effects of pre-damaging by heating and then consolidation were mostly assessed in terms of dynamic elastic modulus (*E_d_*), measured by ultrasound [43,44,60]. In cylindrical samples (15 mm diameter) pre-heated at 200 °C, the *E_d_* (preliminarily artificially decreased to ~30% of its initial value) was completely restored and even enhanced after consolidation by all the following treatments: (i) 0.1 M DAP + 0.1 mM CaCl_2_ in 10 vol % ethanol (applied twice); (ii) 0.1 M DAP + 0.1 mM CaCl_2_ in 10 vol % isopropanol (applied twice); (iii) 1 M DAP + 1 mM CaCl_2_; (iv) 3 M DAP followed by limewater poultice [44]. However, in the case of 20 mm thick slabs, where the *E_d_* had decreased to ~30% of its initial value after accelerated weathering, only treatments (iii) and (iv) were able to fully restore the original *E_d_* [44]. Treatments (i) and (ii) only caused a limited improvement, which might be a consequence of the volatility of alcohols contained in these formulations (likely causing a reduction in the treatment penetration depth) or of the little amount of newly formed CaP, insufficient to bridge the cracks [44].

In prismatic samples (20 mm thick) pre-heated in an oven at 400 °C, the *E_d_* (preliminarily decreased to ~10% of its initial value) was completely restored and even enhanced after treatment with a 3 M DAP solution, followed by a limewater poultice [43]. The penetration depth of the DAP solution was found to be at least equal to the sample thickness (viz., 20 mm) [43]. Compared to the untreated reference, the abrasion resistance was increased by about 10 times on the treated surface and was significantly higher also at 4 mm depth, which indicates a depth of new phase formation at least equal to 4 mm [43].

To reproduce the gradient in properties that is present in naturally weathered marble (i.e., more decay near the surface), samples with differential decay were produced by heating in contact with a hot plate at 200–300 °C for 20 s [65]. The *E_d_* in the first 1 cm from the heated surface (artificially decreased to ~40% of its initial level) was completely restored and even significantly improved (up to +150%) after a single treatment with a 1 M DAP + 1 mM CaCl_2_ solution [38]. The *E_d_* was also completely restored after double application of a 0.1 M DAP + 0.1 mM CaCl_2_ solution in 10 vol % ethanol or isopropanol [38].

The consolidation ability has also been assessed on a naturally weathered sample, which had been exposed outdoors for ~150 years [43]. The sample initially exhibited a decrease in *E_d_* of ~40% in the first 1 cm from the external surface, a decrease of ~10% between 1 and 2 cm and no damage in the underlying part [43]. After treatment with a 3 M DAP solution followed by a limewater poultice, the ~40% loss in *E_d_* was almost completely restored in all parts [43]. Good results were also obtained on this naturally weathered sample in terms of resistance to material loss, assessed by the scotch tape test [43].

Compared to AmOx (frequently used in marble conservation), the phosphate treatment proved to have higher penetration depth [61] and higher effectiveness in re-establishing cohesion among grains [43,44]. In fact, newly formed calcium oxalate is mainly limited to ~1 mm from the treated surface [61]. With respect to ethyl silicate, the phosphate treatment exhibited comparable [44] or higher [43] effectiveness, because the amorphous silica formed by ethyl silicate does not chemically bond to calcite grains [68,69]. In turn, the phosphate treatment has a strong advantage in terms of compatibility, as described in detail in Section 3.6.

### 3.3. Ability to Arrest and Prevent Bowing of Thin Slabs

The ability of the phosphate treatment to arrest bowing of pre-bowed marble slabs was investigated in Reference [44]. Four different formulations of the phosphate treatment were considered: (i) 0.1 M DAP + 0.1 mM CaCl_2_ in 10 vol % ethanol (applied twice); (ii) 0.1 M DAP + 0.1 mM CaCl_2_ in 10 vol % isopropanol (applied twice); (iii) 1 M DAP + 1 mM CaCl_2_; (iv) 3 M DAP followed by a limewater poultice. After 12 thermal cycles (20–90–20 °C), all the investigated treatments demonstrated some ability to reduce bowing, compared to the untreated reference [44]. The reduction in bowing was more pronounced for the treatment formulations based on lower DAP concentrations: while untreated references exhibited average bowing of 0.28 mm/m, samples subjected to treatments (i) and (ii) experienced average bowing of 0.06 and 0.03 mm/m, respectively [44]. This is consistent with the lower reduction in open porosity caused by these formulations [44]. Indeed, limited reduction in open porosity after consolidation is positive in view of reducing the tendency to bow, because the presence of voids between calcite grains allows for some grain deformation upon temperature variations, without causing stress at grain boundaries and without opening of new micro-cracks [70]. However, at the end of the thermal cycles, the residual *E_d_* of samples subjected to treatments (i) and (ii) was similar to that of the untreated references (38–39% of the initial *E_d_* against 31%, respectively) [44]. Samples treated by formulations based on higher DAP concentrations (viz., treatments (iii) and (iv)), although experiencing almost as much bowing as the untreated references, at the end of the thermal cycles exhibited higher residual *E_d_* than the untreated references (50–57% of the initial *E_d_* against 31%, respectively) [44].

Notably, the AmOx treatment (tested for comparison’s sake) was found to dramatically increase bowing of pre-bowed slabs, which after 12 thermal cycles exhibited bowing about 6 times as much that of untreated reference slabs (1.64 mm/m against 0.28 mm/m, respectively) [44]. Ethyl silicate provided results substantially similar to those of the phosphate treatments (iii) and (iv) [44].

The 1 M DAP + 1 mM CaCl_2_ formulation was also tested as a possible treatment to prevent bowing of fresh (i.e., not pre-bowed) marble. Encouraging results were obtained, as after 18 thermal cycles (20–90–20 °C) treated samples exhibited lower bowing than untreated samples (0.30 against 0.52 mm/m, respectively) and higher residual *E_d_* (51% of the initial *E_d_* against 34%, respectively) [44].

### 3.4. Self-Cleaning Ability

**Figure 6:**
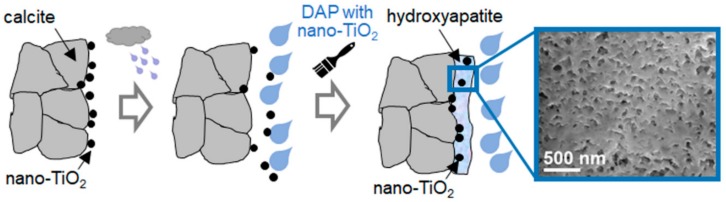
Scheme illustrating nanoTiO_2_ removal by rain when nanoparticles are directly applied onto marble surface. A significant improvement in durability can be obtained by incorporating nanoTiO_2_ in HAP coatings (on the right, a FIB-SEM cross section of a HAP-TiO_2_ nanocomposite is illustrated).

To provide marble with durable self-cleaning ability, the incorporation of photocatalytic TiO_2_ nanoparticles into HAP coatings was investigated in Reference [45]. In fact, nanoTiO_2_ application directly onto architectural surfaces is already frequent practice onsite but nanoparticles are easily washed away by rain because they adhere to the substrate only by weak physical bonding. The limitation of the current practice of nanoTiO_2_ application and the possible improvement represented by nanoTiO_2_ combination with HAP are schematically illustrated in Figure 6.

Two possible routes of nanoTiO_2_ combination with HAP were investigated and compared to treatment with HAP alone and nanoTiO_2_ alone: (i) sequential application of nanoTiO_2_ over an already formed layer of HAP (formed by treatment with a 3 M DAP solution, followed by a limewater poultice); (ii) direct addition of nanoTiO_2_ to the 3 M DAP solution (also in this case followed by a limewater poultice) [45]. By route (ii), HAP-TiO_2_ nanocomposites were obtained, exhibiting Ti-O-P chemical bonds between the TiO_2_ nanoparticles and the HAP matrix [45]. In addition to high consolidating ability (UPV increase from 0.7 to 3.1 km/s), the HAP-TiO_2_ nanocomposites exhibited high photocatalytic activity and high resistance to nanoTiO_2_ leaching by rain, as assessed by accelerated durability tests [45]. The HAP-TiO_2_ nanocomposites demonstrated much better durability than TiO_2_ nanoparticles directly applied onto the marble surface, which were abundantly leached by the simulated rain [45]. Notably, the HAP-TiO_2_ coating exhibited lower cracking and denser microstructure, compared to the HAP coating alone, which was ascribed to the positive effect of nanoparticles, providing seeds for HAP nucleation and reducing shrinkage and cracking during drying [45]. The encouraging results obtained by incorporation of nanoTiO_2_ into HAP coatings were consistent with previous results reported in the biomedical literature, where HAP-TiO_2_ composites were found to have enhanced photocatalytic activity compared to TiO_2_ alone [71,72,73]. Also sequential application of TiO_2_ over an already formed HAP layer (route (i)) was found to provide better durability than nanoTiO_2_ directly applied over marble surface, because the flower-like morphology of HAP and its high specific surface area likely allow for better physical adhesion between the particles and the substrate, compared to direct application onto marble [45].

### 3.5. Anti-Fungal Ability

Products commonly used against biodeterioration (mainly owing to fungi) are not completely satisfying, so the possible use of HAP and ion-substituted HAP to provide stone with anti-fungal ability has been explored [74,75]. The effectiveness of doping HAP with strontium [74], barium [74] and silver [75] ions and silver nanoparticles [75] has been investigated. At present, research has focused on the synthesis and the characterization of the antimicrobial activity of HAP nanoparticles, while the evaluation of the effects of applying so-formed nanoparticles to real stone has not been fully addressed yet.

Strontium- and barium-substituted HAP were produced as follows [74]: (i) HAP (used as reference) was synthesized by adding a 1 M DAP solution to a 1 M Ca(NO_3_)_2_·4H_2_O solution at 80 °C, the pH being constantly maintained at 10; (ii) partially substituted strontium HAP (SrCaHAP) was synthesized as above, using a 0.5 M Ca(NO_3_)_2_·4H_2_O + 0.5 M SrCl_2_·6H_2_O solution; (iii) totally substituted strontium HAP (SrHAP) was synthesized as above, using a 1 M SrCl_2_·6H_2_O solution; (iv) totally substituted barium HAP (BaHAP) was synthesized as above, using a 1 M BaCl_2_·6H_2_O solution. The synthesized materials were pulverized and sprayed as hydroalcoholic suspensions onto “simulated artefacts,” kept in dark and humid environment for 15 days [74]. All the synthesized materials provided some antifungal activity, decreasing in the order SrHAP > HAP > BaHAP > SrCaHAP > blank [74].

Combination of silver and HAP was performed by silver incorporation either during or after HAP synthesis [75], as described in the following: (i) HAP (used as reference) was synthesized as described in the previous paragraph; (ii) partially substituted silver HAP was synthesized as above, using a 0.5 M Ca(NO_3_)_2_·4H_2_O + 0.5 M AgNO_3_ solution; (iii) totally substituted silver HAP was synthesized as above, using a 1 M AgNO_3_ solution; (iv) already formed HAP was added to a suspension of silver nanoparticles (HAP:silver molar ratio of 1:1); (v) already formed HAP was suspended in water at pH 5 containing AgNO_3_ (HAP:silver molar ratio of 1:1). All the silver-containing materials exhibited significant antimicrobial activity, depending not only on the amount of silver but also on its location (on the surface and/or in the pores) [75]. Formulations (ii), (iv) and (v) showed the highest potential [75]. However, the feasibility of applying these treatments to stone artworks still needs to be evaluated, in terms of nanoparticle adhesion to the substrate, nanoparticle resistance to leaching by rain and possible colour change. Following the treatment, the synthesized materials (ii)–(iv) exhibited a progressively darker colour, from pale yellow to brown [75]).

### 3.6. Compatibility

In addition to being sufficiently effective, any treatment intended for marble conservation (i.e., protection, consolidation or functionalization) needs to meet additional compatibility requirements, in terms of aesthetic appearance (Section 3.6.1), microstructural (Section 3.6.2) and physical properties (Section 3.6.3).

#### 3.6.1. Colour Change

The aesthetic compatibility of various formulations of the phosphate treatment has been evaluated by measuring the colour change after treatment (ΔE*), calculated as ΔE* = (ΔL*^2^ + Δa*^2^ + Δb*^2^)^1/2^, where L* = black ÷ white, a* = green ÷ red, b* = blue ÷ yellow are the LABCIE colour parameters [76]. A summary of the colour change detected on marble treated by various formulations is reported in Table 5. Considering that a “just noticeable difference” detectable by human eye is ΔE* = 2.3 [76] and that the colour change commonly accepted after conservation treatments is ΔE* = 5 [66], all the tested formulations can be considered as aesthetically compatible. Indeed, basically in all the cases the colour change after treatment was lower than the human eye detectability threshold (Table 5). With a few exceptions, the phosphate treatment generally causes some brightening (ΔL* > 0) and a shift towards blue (Δa* < 0) and green (Δb* < 0). For comparison’s sake, the AmOx treatment and ethyl silicate were found to cause higher colour changes when applied to marble (ΔE* = 2.5 for AmOx and ΔE* = 12.2 for ethyl silicate) [43].

#### 3.6.2. Porosity and Pore Size Distribution

In terms of microstructural compatibility, the effects of the phosphate treatment on the open porosity and pore size distribution of treated marble have been specifically investigated only in a few studies [43,44], while an indirect evaluation of the alterations in the pore system has been assessed in other studies by measuring the change in water absorption [4,59]. Alterations in open porosity and pore size distribution have been determined by mercury intrusion porosimetry (MIP) on artificially weathered marble [43,44] and naturally weathered marble [43]. In general, the higher the DAP concentration, the higher the reduction in porosity [44]. However, in no case does complete pore occlusion occur, even when high DAP concentrations (e.g., 3 M DAP) are used [43,44]. This is important for ensuring a fair durability to thermal cycles, because the lower the porosity, the higher the sensitivity to temperature variations (cf. Section 3.7.2).

#### 3.6.3. Water and Water Vapour Transport Properties

Variations in water absorption and water vapour permeability after the phosphate treatment have rarely been specifically measured, since these properties are generally very low in marble (because of its low porosity) and since the phosphate treatment was found to cause minor alterations in open porosity and pore size distribution. Slight decreases in water absorption by capillarity and water vapour permeability were assessed in Reference [4] on compacted powder (simulating weathered marble), treated with nanolimes and then a 5% ATP solution. However, because of the nature of the specimens (compacted powder), water absorption and water vapour permeability were unrealistically high before the treatment. In Reference [59], the water vapour permeability after treatment with a 1 g/L monocalcium phosphate solution (forming HAP by a “metasomatic reaction”) was found to be almost unaffected after treatment, so that the “breathability” of the stone was preserved.

### 3.7. Durability

The durability of marble subjected to the phosphate treatment has been investigated with respect to those weathering processes that threaten marble the most: resistance to dissolution in rain [37,38,52,63], resistance to thermal deterioration [44,77,78] and resistance to biodeterioration [62].

#### 3.7.1. Dissolution in Rain

The resistance to dissolution in rain was basically the main parameter investigated in the studies aimed at evaluating the protective ability of the CaP coating [37,38,52,63]. The best resistance to dissolution was demonstrated by the OCP coating formed using a 0.1 M DAP + 0.1 mM CaCl_2_ solution in 10 vol % ethanol, because cracks and pores in the coating were prevented [38]. Coatings formed at higher DAP concentrations (e.g., 1 M DAP + 1 mM CaCl_2_ [52] and 3 M DAP followed by limewater treatment [63]) experienced some deterioration during exposure to simulated rain, because the presence of cracks and pores allowed water to reach the marble substrate, triggering dissolution and causing some detachment of the coating [38]. Nonetheless, when combined with TiO_2_ nanoparticles, the HAP coating formed using a 3 M DAP solution demonstrated good resistance to dissolution in water, likely thanks to the positive action that nanoparticles had on HAP microstructure (reduction of cracks and pores by seeding effect and anti-shrinkage effect) [45].

#### 3.7.2. Thermal Weathering

The resistance to thermal deterioration was investigated by subjecting consolidated marble to accelerated thermal cycles, resembling natural thermal cycles more or less realistically.

In the first study aimed at evaluating the thermal behaviour of consolidated marble, pre-weathered samples were consolidated by a 3 M DAP solution followed by a limewater poultice [77]. Then, consolidated samples were heated at 400 °C for 1 h [77]. After this thermal cycle, only a moderate decrease in *E_d_* was found, which is a positive consequence of the fact that the treatment did not significantly occlude the open porosity in marble [77]. However, because the tested conditions (400 °C for 1 h) were definitely more severe than field conditions, the measured behaviour is not necessarily fully representative of marble behaviour in real conditions, hence more realistic conditions were later evaluated.

The same formulation of the phosphate treatment (3 M DAP followed by a limewater poultice) was tested by subjecting consolidated marble to dilatometric tests in a realistic temperature range (20–90–20 °C) [78]. After treatment, an increase in the thermal expansion coefficient was registered, compared to the untreated reference, as a consequence of the re-established cohesion between calcite grains [78]. Nonetheless, no increase in residual strain was found, compared to the unweathered condition, which was ascribed to ability of newly bonded calcite grains to deform elastically, without experiencing cracking, which in turn was made possible by the limited occlusion of micro-cracks after treatment [78].

A systematic comparison of the resistance to thermal weathering was carried out between four different formulations of the phosphate treatment, AmOx and ethyl silicate [44]. The investigated formulations were: (i) 0.1 M DAP + 0.1 mM CaCl_2_ in 10 vol % ethanol (applied twice); (ii) 0.1 M DAP + 0.1 mM CaCl_2_ in 10 vol % isopropanol (applied twice); (iii) 1 M DAP + 1 mM CaCl_2_; (iv) 3 M DAP followed by limewater poultice. After pre-weathering in an oven and after consolidation, samples were subjected to thermal cycles in a realistic temperature range (20–90–20 °C) in dry and wet conditions (4 dry + 4 wet cycles) [44]. All the phosphate-treated samples exhibited better behaviour than the untreated reference, as they experienced residual strain close to zero (i.e., almost no irreversible damage) and retained higher *E_d_* at the end of the thermal cycles [44]. This was possible thanks to the increased adhesion between calcite grains and to the limited reduction in porosity after treatment [44]. Samples treated by ethyl silicate and, especially, AmOx exhibited much higher residual strain and lower residual *E_d_* after the thermal cycles. This points out the importance of determining the thermal durability of treated marble to evaluate the suitability of any consolidating treatment: right after treatment, ethyl silicate apparently provided higher consolidation than some of the phosphate formulations; however, after the thermal cycles, all the initial advantage of ethyl silicate was lost, because of its much worse resistance to thermal cycles [44]. In the case of AmOx, consolidated samples performed even worse than the untreated references, as they experienced higher residual strain and lower *E_d_* retained [44].

#### 3.7.3. Biodeterioration

The resistance to biodeterioration was investigated because possible residues of unreacted DAP in the stone may favour biological growth, as DAP is rich in phosphorus and nitrogen [62]. In one application to a real case study, some traces of biological growth were actually found in some treated areas (but not in all) 1 year after consolidation with a 1 M DAP solution [46]. However, laboratory studies involving different DAP concentrations have pointed that DAP residues can be successfully eliminated at the end of the treatment by suitable procedures: in samples treated with a 0.76 M DAP solution [35] and a 1 M DAP + 1 mM CaCl_2_ solution [34], DAP residues were completely removed by rinsing with water at the end of the treatment [34,35]; in samples treated with 3 M DAP solutions, DAP residues were successfully removed by application of a limewater poultice, left to dry in contact with the samples [43,51].

In the case of samples treated with a 7% *w*/*v* DAP solution, applied by poulticing for 48 h, some DAP residues were found after rinsing with water by spraying 4 times for 10 s [62]. To prevent biological growth, rinsing with water and biocides proved to be more effective than rinsing with water alone, as expected [62]. The most effective method was found to be introducing the biocides directly into the DAP solution, presumably because in this case a deeper penetration of the biocides and their entrapment in the newly formed CaP are obtained [62].

## 4. Limestone

In the case of limestone, the phosphate treatment has been investigated mainly as a consolidant (Section 4.1) but also as a coupling agent for silicate consolidants (Section 4.2). Similar to the case of marble, for any application on limestone the phosphate treatment must ensure suitable compatibility (Section 4.3) and durability (Section 4.4).

### 4.1. Consolidation

**Figure 7:**
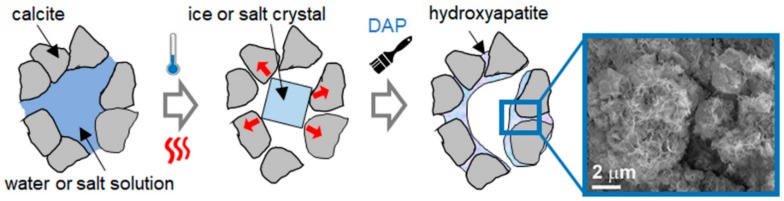
Scheme illustrating the consolidating action of the phosphate treatment, with an example of a SEM image showing newly formed HAP bonding calcite grains in limestone.

The consolidating action of the phosphate treatment on limestone is schematically illustrated in Figure 7.

Besides tests carried out on compacted limestone powder [57] and fresh limestone (Lecce stone, open porosity OP = 30% and *Tuffeau de Maastricht*, OP = 50%) [3], the consolidating effectiveness of the phosphate treatment has been mainly evaluated in limestone after artificial weathering, aimed at reproducing changes in microstructure and mechanical properties similar to those encountered in the field [1,42,48,49]. A summary of the consolidating effectiveness and penetration depth measured on different types of substrate is reported in Table 6.

In the case of artificially weathered Indiana limestone (OP = 15%), after treatment by capillarity for 48 h with a 1 M DAP solution significant increases in mechanical properties were obtained (Δ*E_d_* = +60% and Δσ_t_ = +25%) [1]. On the same kind of stone, an even greater improvement was obtained (Δ*E_d_* = +110%) when 1 mM CaCl_2_ was added to the 1 M DAP solution [48], because the addition of a calcium source favours CaP formation (cf. Section 2.5) [34].

In the case of artificially weathered Globigerina limestone (OP = 40%), a significant consolidating effect (Δ*E_d_* = +47%, Δσ_t_ = +26%, increased resistance to abrasion) was found after consolidation by brushing 10 times with a 3 M DAP solution, followed by a limewater poultice [49]. After treatment, mechanical properties were completely restored to the level before artificial deterioration and even slightly enhanced [49].

In a study aimed at comparing the performance of a 3 M DAP solution applied by brushing, poultice or partial immersion (in all cases followed by application of a limewater poultice), application by poultice and partial immersion were found to be able to fully saturate the samples (25 mm thick) and to cause the greatest mechanical improvement (up to Δ*E_d_* = +50% and Δσ_t_ = +30%) but also the strongest colour change and alteration in pore size distribution [42]. Consequently, application by brushing 10 times was regarded as preferable, because it causes much less pronounced alterations in the pore system, at the cost of only a slight reduction in mechanical efficacy (Δ*E_d_* = +47% and Δσ_t_ = +26%) [42] and penetration depth (18 mm, instead of 25 mm) [51].

It is noteworthy that, in the case of application by brushing, a significant increase in the penetration depth (from 6 to 18 mm) was registered after the end of the application procedure [51]. This was a consequence of the redistribution of the DAP solution in the pore system, as the solution is gradually absorbed in narrower pores [51]. Some consolidant redistribution takes place also during drying, when the solution (containing unreacted DAP) progressively moves toward the surface [51]. During this process, PO_4_^3−^ ions in the solution are progressively consumed to form HAP and more PO_4_^3−^ ions can dissociate from HPO_4_^2−^, so that HAP formation proceeds further and is maximal near the surface [51]. This explains why, even in samples evenly impregnated with the DAP solution (e.g., by capillarity), more abundant formation of HAP is registered near the surface [1,49,51].

When the consolidating efficacy of the phosphate treatment was compared to that of commercial consolidants, AmOx was found to provide higher strengthening in the first 0–2 mm from the surface but no consolidating action below this depth [3]. This is a consequence of the fast reaction between the AmOx solution and the substrate [61]. Considering that the DAP solution is able to penetrate much more in depth (up to 25 mm) and to provide more in-depth consolidation [49,51], the advantage of the phosphate treatment is evident. Unlike the case of marble, ethyl silicate was found to provide more strengthening than the phosphate treatment when they were applied to Globigerina limestone [49]. This is due to the specific mineralogical composition of Globigerina limestone, which contains quarzitic fractions and clays that allow for chemical bonding with the amorphous silica formed by ethyl silicate hydrolysis and condensation [49,69,80]. However, compared to the phosphate treatment, ethyl silicate causes a stronger reduction in open porosity and a bigger increase in the number of smaller pores (cf. Section 4.3.2), which leads to inferior durability to ice or salt crystallization cycles (cf. Section 4.4). Consequently, the initial advantage of ethyl silicate (which causes higher consolidation right after treatment [49]) is lost when treated stone is subjected to durability tests [50].

The effects of the phosphate treatment applied to a porous marlstone in a field study have been recently reported [46], as discussed in detail in Section 6.

In addition to in situ formation of HAP directly inside stone pores (as was the case in all the above-reviewed studies), the use of already-formed HAP nanoparticles has been investigated in several formulations: (i) HAP nanoparticles [81]; (ii) strontium-substituted HAP nanoparticles [81]; (iii) mixtures of HAP and Ca(OH)_2_ [82]; (iv) mixtures of HAP and caoxite (calcium oxalate trihydrate) [83]. All these materials were synthetized and then applied to a chalk stone (a type of limestone) [81,82,83]. In general, an increase in compressive strength and a decrease in water absorption were registered [81,83]. Whereas in the case of the HAP-caoxite mixture an acceptable colour change was found (ΔE* = 1.8) [83], in the case of HAP and strontium-substituted HAP nanoparticles significant whitening occurred after treatment [81], possibly because of low penetration depth of the particles. This makes further optimization of this approach necessary.

### 4.2. Effectiveness as a Coupling Agent for Silicate Consolidants

**Figure 8:**
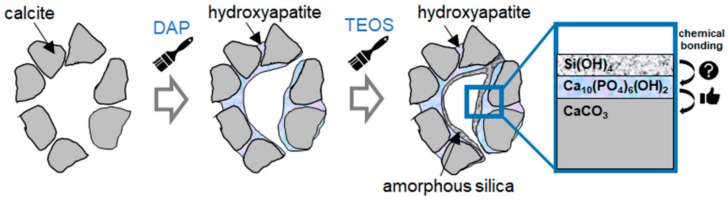
Scheme illustrating the possible use of HAP as a coupling agent for silicate consolidants applied to limestones. However, the bonding between the silicate consolidant and the HAP layer was found to be mechanical in nature.

The possibility of using the phosphate treatment to deposit a layer of CaP that serve as a coupling agent for silicate consolidants was investigated in Reference [48]. The idea was to first cover the internal surface of pores with a layer of HAP, chemically bonded to the calcitic substrate; then, to apply a silicate consolidant, so that the silica gel formed by the silicate consolidant could chemically bond to the HAP layer, thanks to the OH groups present in the HAP formula unit (and not in the calcitic substrate) [48]. A scheme illustrating the rational of this approach is illustrated in Figure 8.

However, FT-IR detected no such chemical bonding between HAP and trimethylethoxysilane (TMES, selected among silicate consolidants because Si-CH_3_ has a characteristic FT-IR band) [48]. Consequently, OH groups in HAP appear not to be available for reaction, which implies that no effective action of HAP as a coupling agent for silicate consolidants can be expected.

From a mechanical point of view, the effectiveness of using HAP as a coupling agent for a consolidant based on tetraethoxysilane (TEOS) was evaluated in Reference [57]. In this case, the TEOS-based consolidant was applied after formation of a HAP layer inside stone pores, by treatment with a DAP solution also containing 0.1 M CTAB (added to favour HAP formation) [57]. Compared to application of HAP alone, the subsequent application of the TEOS-based consolidant caused some further mechanical improvement (however at the cost of a significant colour change and decrease of wettability) [57]. However, because no data were reported in the cited study about the effects of applying the TEOS-based consolidant directly to the limestone (with no HAP layer) [57], it is impossible to derive any conclusion about the possible effectiveness of HAP as a coupling agent for silicate consolidants.

#### Combination of TEOS and Hydroxyapatite

The addition of HAP nanoparticles to TEOS was investigated with the aim of improving the performance of TEOS-based consolidants [84,85].

Nanocomposites based on TEOS, polydimethylsiloxane (PDMS, aimed at providing hydrophobicity) and nanoHAP (aimed at reducing cracking and increasing the surface roughness, thus increasing the contact angle) were investigated in Reference [84]. Compared to treatment with TEOS/PDMS alone, addition of nanoHAP resulted in reduced cracking of the new gel, increased mechanical improvement and increased resistance to accelerated aging, with no reduction in breathability and no significant colour change [84]. However, no advantage of using HAP nanoparticles rather than other nanoparticles was highlighted.

Nanocomposites based on TEOS, amylamine (CH_3_(CH_2_)_4_NH_2_, added as a surfactant and a template to reduce cracking of the gel) and nanoHAP (added considering that HAP is present in protective patinas naturally developed over ancient monuments, which are still well preserved thanks to the good adhesion of these patinas to the substrate) were investigated in Reference [85]. The colloidal consolidant exhibited good penetration depth in porous limestone (>20 mm) and caused significant increases in drilling resistance (+11%) and tensile strength (+11%), compared to the untreated reference [85]. The consolidating performance was higher than that of TEOS/nanoHAP alone (with no amylamine) and that of TEOS/amylamine alone (with no nanoHAP), tested for comparison’s sake [85]. This confirmed the important role of both nanoHAP and amylamine [85]. In terms of microstructure of the hardened consolidant, addition of both nanoHAP and amylamine led to increased uniformity, decreased aggregation and decreased cracking, compared to the gels formed with only one of the two additions [85]. Considering the good mechanical strengthening and water repellency achieved by this treatment, the TEOS-HAP nanocomposite was regarded as highly promising [85].

### 4.3. Compatibility

Similarly to the case of marble, treatments intended for limestone consolidation need to meet several compatibility requirements, in terms of both aesthetic appearance (Section 4.3.1), microstructural (Section 4.3.2) and physical properties (Section 4.3.3).

#### 4.3.1. Colour Change

The colour change of different limestones after the phosphate treatment is reported in Table 7. Whereas in the case of white marble the colour change is generally lower than the “just noticeable difference” detectable by human eye (ΔE* = 2.3 [76]) (cf. Section 3.5), in the case of limestone slightly higher colour changes were reported. This is probably a consequence of limestone being generally darker than marble (as the phosphate treatment was found to generally cause some limited brightening). Although visible to the human eye, the measured colour change is generally lower than the threshold commonly accepted for conservation treatments (ΔE* = 5 [66]). A colour change higher than this threshold was reported for Indiana limestone treated with a 1 M DAP solution for 48 h (ΔE* = 7.6 [1]) but in this stone differences up to ΔE* = 11.5 were reported even among untreated samples [1]. A comparison between different application methods (namely, brushing 10 and 20 times, poultice and immersion) highlighted similar colour changes for the different techniques (ΔE* = 2.8, 3.5, 3.4, 2.2, respectively) [42]. For comparison’s sake, the colour change induced by the AmOx treatment was found to range from ΔE* = 0.5 to 4.0, depending on the limestone type [3], and the colour change induced by ethyl silicate was found to range from ΔE* = 1.3 [49] to 10.1 [86], depending on the limestone type and application method.

#### 4.3.2. Porosity and Pore Size Distribution

**Figure 9:**
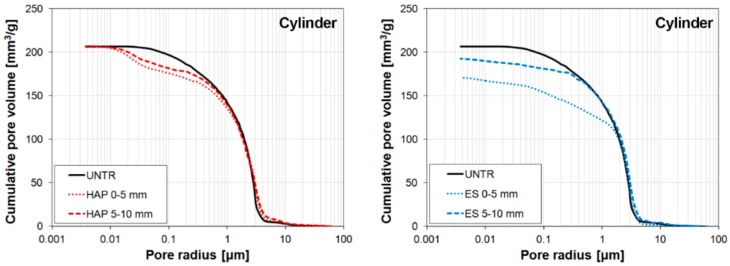
Alterations in pore size distribution of Globigerina limestone treated with a 3 M DAP solution, followed by a limewater poultice (“HAP,” left) and with a commercial ethyl silicate (“ES,” right), in comparison with the untreated reference (“UNTR”). Both consolidants were applied by brushing 10 times cylindrical samples (50 mm height, 20 mm diameter) on their lateral surface. The pore size distribution was determined by mercury intrusion porosimetry (MIP) on samples obtained at increasing depth from the treated surface. The “ES” sample was left to cure for 1 month before testing. Image adapted from Reference [49] with permission from Elsevier.

In terms in open porosity, minor reductions were found after treatment: OP decreased from 14.5% to 13.4% in Indiana limestone treated with a 1 M DAP solution for 48 h [1] and from 37.3% to 35.1% in Globigerina limestone treated with a 3 M DAP solution for 48 h, followed by a limewater poultice [49]. In terms of pore size distribution, after the respective treatments some increase in the relative number of pores with size <0.1 μm [1] and of pores between 0.01 and 0.2 μm [49] was reported. Such alterations in open porosity and pore size distribution were more pronounced near the surface (first 5 mm) and progressively decreased with depth, both in the case of samples treated by brushing on a single face [49] and by capillarity [1]. In the case of Globigerina limestone treated with a 3 M DAP solution, followed by a limewater poultice, the alterations in pore size distributions are illustrated in Figure 9, in comparison with ethyl silicate (causing much more pronounced alterations).

The fact that greater variations in pore size distribution were detected in the case of samples treated by capillarity is notable, because in this case uniform impregnation and, hence, uniform formation of CaP phases would be expected. The presence of such a gradient was later ascribed to the redistribution of the DAP solution taking place in the pores during drying [51]. In fact, PO_4_^3−^ ions from DAP dissociation react with the stone, forming new HAP. As the available PO_4_^3−^ ions are consumed, new ones are formed from HPO_4_^2−^, so that HAP formation proceeds. During drying, the DAP solution progressively moves towards the surface and the PO_4_^3−^ ions are progressively consumed to form HAP, so that more PO_4_^3−^ can dissociate from HPO_4_^2−^ and HAP formation continues, being maximal near the surface [51].

The alteration in pore size distribution is a very important parameter, as it determines limestone durability to ice and salt crystallization cycles. In fact, an increase in the fraction of smaller pores might lead to increased susceptibility to these weathering processes, because the smaller the pore size, the higher the crystallization pressure [19]. However, as described in Section 4.4, specific experimental tests excluded an increase in the susceptibility to ice and salt crystallization after the HAP-treatment, at least in the investigated conditions. Notably, ethyl silicate was found to induce much more pronounced alterations in open porosity (reduction from 37.3% to 30.7%) and pore size distribution (increase in the number of pores with radius <0.01 μm) [49], which contributed to make ethyl silicate-treated stone more susceptible to ice and salt crystallization than HAP-treated and even untreated stone [50].

#### 4.3.3. Water and Water Vapour Transport Properties

A further aspect linked to the alterations in the pore system is the change in water and water vapour transport properties after treatment, which also depends on the possible changes in the surface wettability. Some reduction in water absorption might be desirable, as water triggers and/or worsens many deterioration processes. However, it is essential that the ability of stone to exchange water and water vapour with the environment be preserved after treatment, to prevent possible issues deriving from water being trapped behind an impermeable consolidated layer [49].

The sorptivity (i.e., the rate of water absorption) of Indiana limestone treated with a 1 M DAP solution for 48 was reduced by ~40% when the treatment was applied by capillarity [1] and by ~20% when the same treatment was applied by brushing [79], likely because of the smaller amount of CaP deposited in the pores in the latter case. The reduction in sorptivity was a consequence of the reduction in coarser pores but for both application methods the total water uptake was almost unchanged [1,79]. Addition of 1 mM CaCl_2_ to the 1 M DAP solution led to a further reduction in sorptivity [48], as a result of the increased amount of CaP phases formed in the presence of an external calcium source [34]. Similarly, double application of a 1 M DAP + 1 mM CaCl_2_ solution caused greater reduction in sorptivity than single treatment [48]. Nonetheless, in all cases the final total water uptake was only slightly diminished [48].

**Figure 10:**
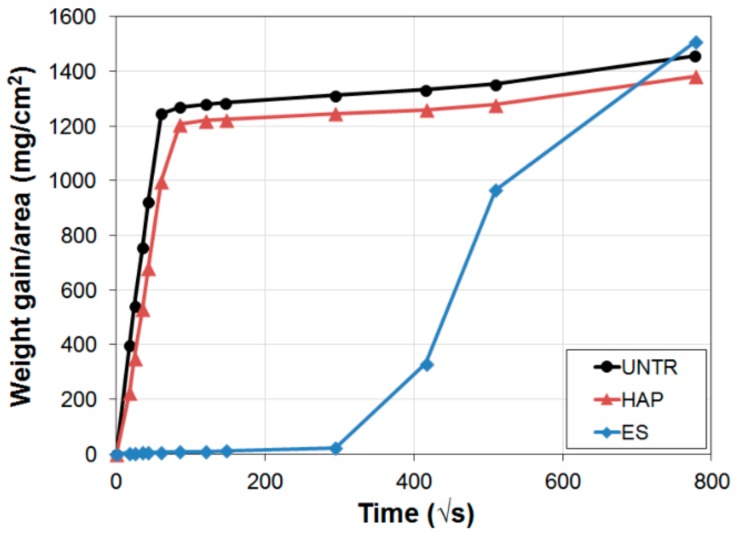
Alterations in water sorptivity of Globigerina limestone treated with a 3 M DAP solution, followed by a limewater poultice (“HAP”) and with a commercial ethyl silicate (“ES”), in comparison with the untreated reference (“UNTR”). Both consolidants were applied by brushing 10 times cubic samples (50 mm side) on a single face, through which water was then let penetrate by capillarity. The “ES” sample was left to cure for 1 month before testing. Image reprinted from Reference [49] with permission from Elsevier.

Minor reductions in sorptivity and water absorption after 24 h were found also in the case of Globigerina limestone treated with a 3 M DAP solution, followed by a limewater poultice [42,49]. In this case, an increase in the contact angle was registered (from 32° to 74–81°, depending on the application method), which may contribute to the measured alterations in water transport properties [42]. However, in no case were dramatic alterations registered, as illustrated in Figure 10.

In Lecce stone (OP = 30%) treated with a 5% DAP solution at pH 8 and treated with 5% ADP solutions at pH 5.6–6 and pH 7–8 for 4, 8 or 17 h, reductions in water uptake by capillarity after 30 min ranged from 32 to 54% [3]. The same treatments applied to *Tuffeau de Maastricht* (OP = 50%) caused reductions of 2–5% [3].

In addition to its modest effect on the exchange of liquid water between the treated stone and the environment, the phosphate treatment was also found to cause minor changes in the drying rate and in water vapour permeability, which is important to ensure good resistance to ice and salt crystallization cycles [49].

For comparison’s sake, limestone treated by AmOx exhibited decreases in water absorption more pronounced than in the case of the phosphate treatment (depending on the initial porosity of the stone, 52–86% and 2–63%, respectively) [3]. This is a consequence of calcium oxalate being formed mainly near the exterior surface, which in turn is a consequence of the very fast reaction between the AmOx solution and the stone [3,61]. In the case of ethyl silicate, a much more drastic alteration in water transport properties is present [49]. In fact, ethyl silicate leaves the treated stone hydrophobic for a long time (at least 1 month according to the products technical data sheets but actually up to 6 months and more [21,87]). During this period, no conservation treatments involving water can be carried out and, most of all, if a water source (e.g., rising damp) is present behind the consolidated layer, the detachment of the hydrophobic layer may occur [49,50]. To restore the wettability of stone treated by ethyl silicate, curing of ethyl silicate can be accelerated by application of a water poultice [87] or a water/alcohol solution [48]. The fact that stone wettability is maintained and that pores are not significantly occluded after the phosphate treatment also ensures retreatability: that is, the DAP-treated stone can be further treated with either the same consolidant or a different one [49].

### 4.4. Durability

The durability of limestone subjected to the phosphate treatment has been investigated for those weathering processes that threaten limestone the most: resistance to freeze-thaw cycles and salt crystallization cycles [50]. Moreover, considering that highly soluble CaP phases may form alongside HAP, with detrimental effects on the treatment performance, the resistance to wetting/drying cycles has been investigated as well [1,50].

#### 4.4.1. Wetting-Drying Cycles

In the case of Indiana limestone treated with a 1 M DAP solution, after 5 wetting/drying cycles some minor decreases in mechanical properties were registered (Δ*E_d_* = −15%, Δσ_t_ = −7%), consistently with the presence of some soluble phases indicated by EBSD [1]. When a 3 M DAP solution, followed by a limewater poultice, was used for Globigerina limestone consolidation, after 7 wetting/drying cycles basically no decrease in mechanical properties was found, in accordance with the lack of any soluble phase indicated by FT-IR [50].

#### 4.4.2. Freeze-Thaw Cycles

**Figure 11:**
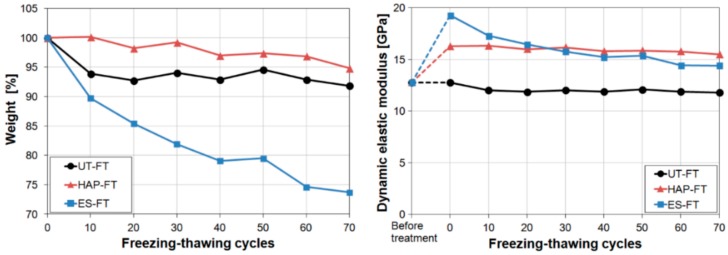
Variations in weight (**left**) and *E_d_* (**right**) of Globigerina limestone subjected to freeze-thaw cycles (FT), untreated (“UNTR”) and treated with a 3 M DAP solution followed by a limewater poultice (“HAP”) and with a commercial ethyl silicate (“ES”). Before testing, the “ES” samples were left to cure for 1 month, then the residual hydrophobicity was eliminated by application of a water poultice [87]. Image reprinted from Reference [50] with permission from Elsevier.

The durability to freeze-thaw cycles of Globigerina limestone treated with a 3 M DAP solution, followed by a limewater poultice, was investigated in Reference [50]. After 70 freeze-thaw cycles, the DAP-treated samples underwent only minor decreases in mechanical properties (Δ*E_d_* = −5%, Δσ_t_ = −6%) [50]. Notably, samples treated by ethyl silicate (tested for comparison’s sake) underwent much higher losses in weight and *E_d_*, as illustrated in Figure 11. This was a consequence of the more pronounced alteration in pore size distribution and the increase in the percentage of small pores caused by ethyl silicate, unlike the phosphate treatment [50]. As a result, samples treated by ethyl silicate underwent higher decreases in tensile strength at the end of the cycles (Δσ_t_ = −24%) and one sample broke after 47 cycles [50] (all the DAP-treated samples survived the tests with no failures).

#### 4.4.3. Salt Crystallization Cycles

**Figure 12:**
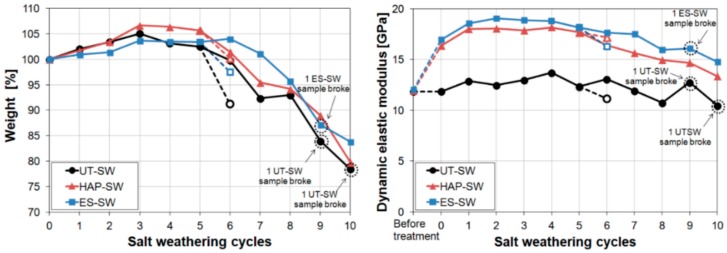
Variations in weight (**left**) and *E_d_* (**right**) of Globigerina limestone subjected to salt crystallization cycles (SW), untreated (“UNTR”) and treated with a 3 M DAP solution followed by a limewater poultice (“HAP”) and with a commercial ethyl silicate (“ES”). Before testing, the “ES” samples were left to cure for 1 month, then the residual hydrophobicity was eliminated by application of a water poultice [87]. Open symbols indicate values of samples desalinated after the 5th cycle. Image reprinted from Reference [50] with permission from Elsevier.

The durability to salt crystallization cycles of Globigerina limestone treated with a 3 M DAP solution, followed by a limewater poultice, was investigated in Reference [50]. After 5 cycles and desalination, basically no decrease in *E_d_* and only Δσ_t_ = −15% were registered [50]. Samples treated by ethyl silicate (tested for comparison’s sake) experienced higher deterioration, as illustrated in Figure 12, leading to Δσ_t_ = −42% after 5 cycles [50]. Moreover, whereas all the HAP-treated samples reached the end of the cycles without any failure, two untreated samples and one ethyl silicate sample failed during the salt crystallization test [50].

## 5. Other Substrates

Following the first studies on carbonate stones, the feasibility and the effectiveness of using HAP and other CaP for the conservation of a variety of different substrates have been explored, as described in the following paragraphs.

### 5.1. Sandstone

The performance of the phosphate treatment has been investigated on sandstones with different amounts of carbonate fractions. In Reference [6], a calcareous sandstone (*Giallo Siena*, CaCO_3_ = 84 wt %) and a siliceous sandstone (*Pietra Serena*, CaCO_3_ = 12 wt %) were treated with a 1 M DAP solution for 48 h, after preliminarily artificial weathering by heating. The increase in mechanical properties following formation of new CaP phases was found to be higher in the calcareous sandstone (Δ*E_d_* = +25%, Δσ_t_ = +24%) than in the siliceous one (Δ*E_d_* = +15%, Δσ_t_ = +18%), as expected [6]. Nonetheless, even in the siliceous stone, a significant improvement was possible, thanks to the fact that in this stone the cement bonding the quarzitic grains has a calcitic composition [6]. Even though in the cited study the consolidating action of the newly formed CaP phases was found to be directly correlated with the available CaCO_3_ content [6], the external addition of a calcium source directly into the DAP solution [34] is expected to overcome any limitation deriving from the amount of calcium ions available in the substrate.

An alternative “biomimetic” method was investigated in Reference [5] on a simulated sandstone, prepared by compacting CaCO_3_ powder and sand. After pre-treatment with nanolimes and treatment with a 10% TAP solution, HAP was found to form [5]. This consolidation substantially increased the compressive strength of the simulated sandstone (σ_t_ increased from <0.05 to 2.5 MPa after treatment), without significantly altering water absorption and water vapour permeability [5]. However, because of the nature of the tested specimens (compacted powder), before treatment the simulated sandstone had basically no cohesion and unrealistically high water and water vapour transport properties.

### 5.2. Salt-Bearing Stone

In real field conditions, stone needing consolidation is rarely uncontaminated (as was the case of samples used in the laboratory studies reviewed in the previous paragraphs). On the contrary, in the field, stone is often affected by the presence of salts, which are dissolved in ground water and are transported into porous substrates by rising damp. Therefore, the effects of applying the phosphate treatment on salt-bearing stone has been preliminarily investigated in Reference [88]. A highly porous limestone (Lecce stone) was contaminated with sodium sulphate decahydrate (Na_2_SO_4_·10H_2_O) by repeated cycles of impregnation with a 14 wt % aqueous solution of Na_2_SO_4_·10H_2_O and drying. Two contamination levels were considered: a higher level (~1.4 wt % SO_4_^2−^), obtained by performing 2–5 impregnation cycles (the number depending on the sample size and shape) and a lower level (~0.2 wt % SO_4_^2−^), obtained from the previous level after desalination by poulticing [88]. The lower contamination level was considered as representative of stone condition in the field, after desalination that usually should precede consolidation. After contamination with salts and consolidation with a 3 M DAP solution, followed by application of a limewater poultice, abundant formation of new CaP phases was found by FT-IR and SEM-EDS [88]. The number of new phases was found to be higher for higher amounts of salts initially present in the stone pores, likely because the salts increase the sites available for CaP nucleation [88]. Some difference in the composition of the new CaP phases was found by FT-IR: while HAP seems to form when no or low salt contamination is present, OCP seems to form when many salts are present in the pores [88]. This was speculatively ascribed to the effect that foreign ions (such as Na^+^) may have on the composition, crystallinity and solubility of the new CaP phases [88]. Notably, no soluble CaP phases nor phosphate salts were detected by FT-IR [88]. Moreover, after consolidation the amount of sulphates was found to be significantly diminished (~0.02 wt % SO_4_^2−^), likely because the limewater poultice helped remove soluble salts. New CaP phases were found both near the surface and deep inside the samples, which indicates that the presence of salts inside the pores did not prevent penetration of the DAP solution deep into the substrate [88]. In terms of mechanical consolidation, samples that had been preliminarily contaminated, then desalinated and consolidated, were subjected to further salt crystallization cycles, to assess the durability of consolidated stone to further salt weathering. After 10 salt crystallization cycles, DAP-treated samples experienced a 12% decrease in *E_d_*, whereas untreated samples and samples treated by ethyl silicate (tested for comparison’s sake) both underwent a 22% decrease [88]. Based on these preliminary results, the presence of salts inside the pores, when stone is subjected to the phosphate treatment, does not appear to be a major obstacle for a successful treatment outcome.

### 5.3. Sulphated Stone

Carbonate stones and, in general, carbonate substrates (e.g., lime renders) may be affected by formation of a surface layer of gypsum (the so-called “sulphation” phenomenon), as a consequence of the high SO_x_ concentrations experienced in the atmosphere in the past decades: in the presence of humidity, SO_x_ reacts with calcite in the substrate, with consequent gypsum formation.

**Figure 13:**
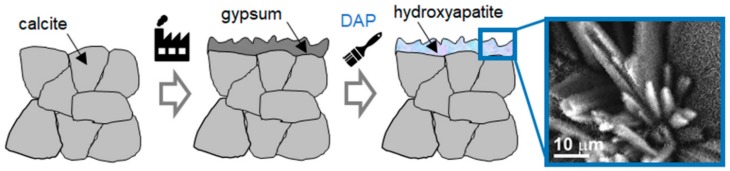
Scheme illustrating the de-sulphating action expected from the phosphate treatment.

The idea of transforming gypsum into less soluble calcium phosphate by the phosphate treatment is schematically illustrated in Figure 13. Similarly to the case of calcite, conversion of gypsum into CaP has been widely investigated in the biomedical field (see for instance [89,90,91]) but specific treatment conditions (in terms of pH, temperature, etc.) are needed for monument conservation.

Studies on the use of phosphate solutions to transform gypsum into CaP as a route for desulphation have been carried out on a variety of artificially sulphated substrates, namely lime paints over stone [7], limestone [9] and marble [8,10]. In all cases, treatment of the gypsum crust with DAP solutions may raise some concerns, because, according to the following reaction (reported in the biomedical literature), H_2_SO_4_ is formed, which might induce corrosion of the underlying carbonate substrate [89]:

10CaSO_4_∙2H_2_O + 6(NH_4_)_2_HPO_4_ → Ca_10_(PO_4_)_6_(OH)_2_ + 6(NH_4_)_2_SO_4_ + 4H_2_SO_4_ + 18H_2_O. (2)

However, it was verified experimentally in Reference [10] that, during reaction between artificially sulphated marble and a 0.1 M DAP solution (containing or not 30 vol % ethanol), the pH never decreased below 8, so the risk of stone corrosion is not realistic.

In the first study about desulphation by conversion of gypsum into CaP [7], sulphated lime paints were simulated by adding gypsum powder to lime paints, applied over a sandstone. So-produced samples were then treated for 48 h by different ammonium phosphate salts (namely, DAP, ADP and TAP) and the composition and the morphology of the newly formed CaP were investigated by XRD and SEM [7]. After treatment with the phosphate solution, a second poultice was applied to remove ammonium sulphate.

According to the following two reactions, gypsum treatment with DAP solutions may lead to formation of two different CaP, namely HAP and brushite [7]:

5CaSO_4_∙2H_2_O + 3(NH_4_)_2_HPO_4_ + 4NH_3_ → Ca_5_(PO_4_)_3_(OH) + 5(NH_4_)_2_SO_4_ + H_2_O; (3)

CaSO_4_∙2H_2_O + (NH_4_)_2_HPO_4_ → CaHPO_4_∙2H_2_O + (NH_4_)_2_SO_4_. (4)

By treating the gypsum-containing lime paints with a 3.8 M DAP solution (pH 9), very fine crystals of HAP were formed, consistent with reaction 3. However, the HAP layer was affected by macroscopic cracks, likely developed because of shrinkage during drying [7].

By treatment with a 2.2 M ADP solution (pH adjusted to 6.5), a compact and dense layer of brushite was obtained [7], according to the reaction:

CaSO_4_∙2H_2_O + NH_4_H_2_PO_4_ → CaHPO_4_∙2H_2_O + (NH_4_)_2_SO_4_. (5)

When a 1.7 M TAP solution (pH adjusted to 11) was used, HAP was formed according to reaction 6 but again the formed HAP layer was macroscopically cracked [7]:

5CaSO_4_∙2H_2_O + 3(NH_4_)_3_PO_4_ + NH_3_ → Ca_5_(PO_4_)_3_(OH) + 5(NH_4_)_2_SO_4_ + H_2_O. (6)

Considering the risk of detachment of the paint layer, because of crack formation after treatment with DAP and ATP, treatment with ADP was identified as the most promising route and selected for field testing in the portal of a church in Nuremberg (XIV-XIX century) [7]. Field testing confirmed the promising results obtained in the lab [7].

In the case of sulphated limestone, the possibility of performing cleaning and consolidation by a single treatment was investigated in Reference [9]. The idea was to take advantage of the calcium ions dissolved from the superficial gypsum crust to favour CaP formation inside the underlying stone [9]. The proposed reaction for gypsum transformation into HAP was the following [9]:

10CaSO_4_∙2H_2_O + 10(NH_4_)_2_HPO_4_ + OH^−^ → Ca_10_(PO_4_)_6_(OH)_2_ + 10(NH_4_)_2_SO_4_ + H_2_O + 4HPO_2_^−^_(aq)_. (7)

A calcarenite (*Arenisca Ronda*, OP = 17%) was artificially sulphated by immersion in 1 M H_2_SO_4_ for 2 h, which led to formation of a gypsum crust with 30–80 μm thickness [9]. The influence of several reaction parameters (viz., DAP concentration ranging from 0.5 to 3 M and application time ranging from 10 to 240 min) on the new phase composition and morphology was investigated and changes in phase composition were monitored up to 6 months after the treatment [9]. After application of a 3 M DAP solution for 60 min, gypsum was completely removed and new CaP phases were formed, including HAP, brushite and OCP, as well as some residual DAP and ammonium sulphate (the latter two effectively removed by washing) [9]. Phase formation was proposed to follow Ostwald’s rule, that is, the phase with the fastest precipitation rate is preferentially formed, even if it is not the most stable phase [9]. Hence, more soluble brushite is kinetically favoured over less soluble HAP because of the lower interfacial energy (and hence the lower nucleation energy) between the phase and the solution [9]. Phase evolution was found to continue after the end of the treatment application and apparently was completed after 1 week [9]. The film became increasingly continuous and homogeneous after washing to remove unreacted DAP, as new precipitation of CaP took place in the fissures [9]. Notably, depending on the treatment conditions, the new CaP layer exhibited diffused cracking and detachment of fragments, while a fissure (30–35 μm thick) was present between the CaP crust and the substrate [9]. Thanks to formation of CaP phases, mechanical strengthening was revealed by increased micro-drilling resistance (MDR) down to a depth of ~4 mm from the treated (sulphated) surface [9].

In the case of sulphated marble, the possible desulphation by treatment with DAP solutions was investigated in Reference [8] and [10]. In Reference [8], white marble was artificially sulphated by reaction with sulphur dioxide in a climatic chamber. Treatment with a 0.02 M DAP solution for 5 days led to complete transformation of gypsum into HAP (assessed by XRD) [8]. The HAP layer was reportedly not cracked but the capillary water absorption of marble was almost unaffected by the treatment [8], which suggests that the HAP layer was porous. The adhesion between the HAP layer and the marble substrate was reportedly good, because no clear interface between the HAP layer and the substrate was visible [8]. The treatment caused a minor colour change (ΔE* = 1.5) [8], below the human eye detection limit (ΔE* = 2.3 [76]).

In Reference [10], artificially sulphated samples of Carrara marble were produced by immersing marble in H_2_SO_4_ at pH 2 for 24 h. After treatment for 24 h with a 0.1 M DAP solution containing 30 vol % ethanol (the same formulation developed for gypsum stuccos, see Section 5.4), a new CaP phase was formed, having morphology resembling that of HAP [10]. However, a conclusive identification was not possible by grazing incidence XRD, likely because of the small amounts of new phases formed over the gypsum crystals [10].

For all the sulphated substrates, it should be noted that, if dust and particulate matter are embedded in the gypsum crust (thus forming the so-called “black crusts”), transformation of gypsum into CaP might render the black crust more resistant to dissolution and also more resistant to cleaning. Therefore, whenever possible, it is recommended to remove the black crust before application of the phosphate treatment. A further aspect that should be taken into account is that the interface between the gypsum layer and the underlying stone might be weak, so a preliminary evaluation should be carried out to assess the feasibility of desulphation by the phosphate treatment.

### 5.4. Gypsum Stuccoes

Stuccoes are decorative elements made of gypsum-based pastes and mortars, usually designed to imitate marble. Because of the high solubility of gypsum in water (~2.4 g/L), gypsum stuccoes exposed outdoors are subject to dissolution in rain or rising damp, with consequent surface recession and pulverization. Traditional consolidants (such as ethyl silicate and organic products) exhibit limitations when applied to gypsum stuccoes [92], therefore treatment with DAP solutions has been explored [10].

According to the reactions proposed in the literature to describe the transformation of gypsum into CaP by reaction with DAP solutions (cf. Section 5.3), either HAP (reactions 2, 3 and 7) and/or brushite (reaction 4) may be formed. In the case of simple gypsum powder immersed in a 1 M DAP solution, gypsum was found to be converted to brushite almost entirely after 1 day and entirely after 28 days [46].

In the case of hardened gypsum pastes simulating stuccoes, the influence of the DAP concentration (0.05 to 0.5 M), of the addition of ethanol to the DAP solution (0 to 50 wt %) and of the solution pH (8 or 10) on the composition and the microstructure of the new CaP phases was investigated in Reference [10]. By increasing the ethanol concentration (thus reducing gypsum solubility and hence the amount of calcium ions available for reaction), by increasing the DAP concentration or by increasing the pH, formation of the most stable CaP phase (i.e., HAP) can be obtained [10]. However, even in a pH range and for a starting Ca/P ratio such that formation of HAP should be favoured, brushite precipitation was found to be kinetically favoured, because brushite requires HPO_4_^2−^ ions (by far the most abundant species originated from DAP dissociation) while HAP requires PO_4_^3−^ ions (which are present only in very minor amounts) [10].

Alongside the composition and the solubility of the new CaP phases, the possible presence of cracks in the new CaP layer is also an important parameter, for reducing the gypsum stucco dissolution in water.

The HAP layers obtained at high DAP concentration (0.5 M), high ethanol addition (50 wt %) and high pH (10) were diffusedly cracked, presumably because of excessive growth of the HAP layer [10]. On the contrary, brushite formed by reacting gypsum pastes with a 0.1 M DAP solution containing 30 vol % ethanol at pH 8 was uncracked [10]. Consequently, the protective and consolidating performance of this latter formulation was tested. Being less soluble than gypsum (although more than HAP), uncracked brushite was able to reduce the weight loss of gypsum stuccoes in water by 17% and to increase their dynamic elastic modulus *E_d_* by 3%, their static elastic modulus *E* by 5% and their tensile strength Δσ_t_ by 13% [10]. The performance of the treatment was limited by the reduced depth of brushite formation (~100 μm), owing to the fast reaction between gypsum and the DAP solution (notwithstanding the addition of ethanol to reduce gypsum solubility) [10].

Slightly higher mechanical improvement (Δ*E_d_* = +5%, Δσ_t_ = +16%) was obtained by reacting gypsum with a more concentrated DAP solution (1 M DAP) [11]. However, such a small mechanical benefit was achieved at the cost of formation of a much higher amount of ammonium sulphate (NH_4_)_2_SO_4_, formed as a by-product according to reactions 2–4 and 7. Being water soluble, ammonium sulphate can be easily removed but this implies a second treatment after impregnation with the DAP solution. Moreover, when gypsum was treated with a 1 M DAP solution (with no ethanol addition), koktaite ((NH_4_)_2_Ca(SO_4_)_2_∙H_2_O) was also formed, likely because of an excess of calcium ions in the solution [11]. Koktaite is water soluble, so it can be removed together with ammonium sulphate but again this requires a further step. Preventing koktaite formation by ethanol addition to the DAP solution, by reducing gypsum solubility, was found to be an effective strategy, as no koktaite was found when ethanol was added [10].

### 5.5. Concrete

In the case of ordinary Portland cement (OPC), formation of a surface layer of “biogenetic” HAP (i.e., HAP produced through the action of living organisms) was investigated in Reference [12]. The method is based on HAP formation from the combination of calcium ions from the OPC pore solution, a phosphate pH buffer and the metabolic processes of the bacteria [12]. Samples of hardened OPC pastes (water/cement ratio = 0.4) were immersed in a solution at initial pH 6.2 containing LB broth (a medium used for the growth of bacteria), KH_2_PO_4_ and K_2_HPO_4_, and were inoculated with the bacterium *Pseudomonas fluorescens* [12]. After incubation for 20 days, a layer of poorly crystalline, nanometric, carbonated HAP was formed (assessed by XRD) [12]. In the case of control samples, which had been immersed in the same solution but without inoculation with the bacterium, brushite was found [12]. This was ascribed to the fact that, in the case of the control samples, the solution pH shifted from 6.2 to 7.4, because of Ca(OH)_2_ leaching from the pore solution [12]. This pH value is too low for HAP precipitation, hence brushite was formed [12]. In the case of the samples inoculated with the bacterium, the pH shifted from 6.2 to 9.1, because of the bacterial metabolic processes, which led to precipitation of HAP [12]. The same mechanism of “biogenic” HAP formation is expected to occur also in the case of carbonate stones, where dissolution of the substrate itself is providing the calcium ions necessary for the reaction [12].

### 5.6. Archaeological Wall Paintings

The performance of the phosphate treatment on wall paintings was investigated in Reference [13]. Mortar specimens simulating wall paintings were produced using Ca(OH)_2_ as binder and marble dust or quarzitic sand as aggregates. After curing for 6 months, the samples were treated with 1 M and 2 M DAP solutions, applied by poultice for 3 or 6 h [13]. As a result, porous CaP phases were formed, likely consisting of carbonated HAP (as assessed based on morphology, because no conclusive identification was obtained by XRD) [13]. The new phases were found down to a depth of 20 mm from the treated surface. In the case of the 2 M DAP treatment, some excessive whitening on the sample surface was observed, while in the case of the 1 M DAP treatment insignificant colour change occurred [13]. After treatment, a reduction in material loss by scotch tape test was registered, as well as 8–11% reduction in water sorptivity [13]. The results were regarded as highly promising, which motivates the prosecution of the study on wall paintings to investigate further aspects in the future, such as the interaction between the DAP solution and pigments [13].

### 5.7. Archaeological Bones

The possible use of the phosphate treatment for the conservation of archaeological bones has also been investigated [14,15]. Bones are made of 20–30 wt % collagen, 60–70 wt % minerals (mainly, small crystals of carbonated HAP, containing variable amounts of fluorine, sodium, magnesium and strontium substituting for calcium, hydroxyl or phosphate ions) and 10 wt % water [14]. Archaeological bones are subject to deterioration mainly because of decomposition of collagen, chemical deterioration of the mineral phase and biodeterioration, resulting in pulverization and cracking [14,15]. Traditional polymeric consolidants have shown limitations when applied to bone conservation (e.g., low penetration, embrittlement, shrinkage, yellowing, biodeterioration), while alkoxysilanes have been investigated only recently [14,15]. Therefore, the use of HAP for bone conservation has been explored, considering the very good compatibility between the substrate and the new mineral formed after treatment [15].

The effects of treating bone flour, modern bone and archaeological bone (about 3000 years old) with DAP solutions (0.5, 1 and 2 M) have been investigated in Reference [14]. The DAP solutions were applied by poultice for 2 h (in the case of the 1 M and 2 M solutions, small amounts of ethanol were added to favour penetration into the bone flour) and then cured for 2 weeks. After treatment, HAP was identified by XRD as the only new phase [14]. Porosity and water absorption were diminished, the variation being directly proportional to the DAP concentration, which led to identify the 0.5 and 1 M concentrations as the most suitable ones. After treatment with these concentrations, modern and archaeological bones exhibited increased cohesion, no significant changes in surface topography and texture and no pore occlusion [14].

In Reference [15], pre-treatment of bones with a 10 g/L Ca(OH)_2_ suspension, applied as a calcium source before application of a 5% (~0.3 M) DAP solution for 72 h, was investigated on artificially weathered bones (calcined at 900 °C to remove collagen). At the end of the treatment, HAP (identified by XRD) was formed, which led to a significant increase in compressive strength, without a significant colour change and with only a minor decrease in water absorption [15].

Notably, when both modern and ancient bones were treated with DAP solutions, isolated crystals of magnesium ammonium phosphate (struvite, NH_4_MgPO_4_·6H_2_O) were detected alongside HAP [14]. This was attributed to the presence of some minor amounts of magnesium [14] (Mg in biological apatites can reach ~7% [93]). Magnesium is known to interfere with HAP formation, by associating with phosphate ions (forming magnesium phosphate Mg_3_(PO_4_)·8H_2_O and struvite NH_4_MgPO_4_·6H_2_O) and by slowing down the kinetics of transformation of ACP into crystalline HAP [14]. Consequently, the crystallinity and the size of HAP crystallites formed in the presence of even a very small amount of magnesium are strongly reduced [93]. This is important, because the interference of Mg in HAP formation may limit or impede successful application of the phosphate treatment to a variety of magnesium-rich substrates, such as ivory and also dolomitic marble.

### 5.8. Paper

The possible use of already-formed HAP nanoparticles for the conservation of ancient paper has been investigated in Reference [16]. Paper is mainly composed of cellulose fibres forming a three-dimensional structure and it may also contain hemicellulose, lignin and additives (e.g., binding materials, inorganic fillers, dyes, pigments, metal ions). Paper is subject to aging because of acid substances, moisture, atmospheric O_2_ and oxidative agents, light and micro-organisms, which induce depolymerization of cellulose fibres (promoted by acid pH) and yellowing [16]. Currently used methods for paper conservation include Ca(OH)_2_ and Mg(OH)_2_ nanoparticles, aimed at paper deacidification, but they are subject to carbonation over time [16]. Therefore, the use of hydroalcoholic suspensions of HAP nanoparticles was explored [16]. The nanoHAP suspension was applied to paper from two old books by spraying, brushing or impregnation on both sides of the paper samples, with and without pre-washing. After nanoHAP application, microscopic surface imperfections were covered, pH was increased, yellowing was decreased and a better performance was registered in an accelerated test of light aging [16]. These results indicate the potential of using HAP for paper conservation.

## 6. Field Studies

A first step towards assessment of the performance of the phosphate treatment in real conditions was the treatment of a real marble specimen, coming from a marble gravestone that had been exposed in the field for some 150 years [43]. After treatment with a 3 M DAP solution for 48 h, followed by application of a limewater poultice, only HAP was found as the reaction product [43]. The same treatment, applied onto artificially weathered but uncontaminated samples, led to formation of different phases (mainly OCP). The difference in phase composition was ascribed to the fact that, in the naturally weathered sample, the presence of some gypsum residues on the treated surface might have increased the availability of calcium ions for the reaction; this, combined with the high pH of the subsequent limewater poultice, might have altered phase formation [43]. In addition, the higher surface roughness (owing to exposure in the field for 150 years) might have favoured HAP formation [43]. Newly formed HAP was found to significantly improve the mechanical properties of weathered marble, as assessed by ultrasound and the scotch tape test. In particular, the *E_d_* (which had decreased by ~40% in the first 1 cm from the external surface and by ~10% between 1 and 2 cm) after treatment was basically brought back to its initial value [43]. Such consolidating action was achieved without causing significant pore occlusion and with only a modest colour change (ΔE* = 2.7), visible by the human eye but still lower than the threshold (ΔE* = 5 [66]) commonly accepted for conservation treatments [43].

Several applications of the DAP-based treatment to real buildings and monuments have been reported in Reference [62], including marble and limestone architectural elements and sculptures in Italy, Turkey and in the Vatican City. In general, positive results were found, although detailed data have been reported in the international literature only in a few cases. In the case of a XIX century building in Milan (Italy), made of *Gallina* limestone affected by sulphation, the results of the treatment with a 7% DAP solution, applied by poultice for 20 h, have been reported in Reference [94]. One area of the building, treated only with the DAP solution, exhibited abundant and well distributed phosphorus presence in the first 60–70 μm from the treated surface [94]. Phosphorus was detected down to a depth of ~3 mm but its distribution was found to be inhomogeneous [94]. In general, CaP formation was thought to be favoured by the supply of calcium ions by the gypsum layer. In other areas of the same building, where a Ba(OH)_2_ solution was applied for desulphation before DAP application, a thin layer (~3 μm thickness) containing barium, sulphur and phosphorus was formed. Underneath this layer, phosphorus was found in decayed areas (normally in the microcrystalline matrix between clasts of calcite) down to a depth of 6 mm [94].

A systematic evaluation of the effects of the phosphate treatment application to a rock-cut chamber tomb in Cyprus, made of a powdery porous marlstone, has been recently reported in Reference [46]. After treatment with a 1 M DAP solution for 3 h by poulticing, HAP was found to be the only reaction product detected by XRD and FT-IR. The presence of some residual unreacted DAP (in concentration below the XRD and FT-IR detection limit) was also likely present, because formation of HAP continued after removal of the poultice, leading to more defined FT-IR bands and a denser coating after 1 year. In some areas where gypsum was originally present, formation of brushite was found. No significant difference in the treatment reaction product was detected between areas that had been desalinated before DAP application and areas that had not. One year after treatment, some traces of biological growth were found in some treated areas but not in all of them, so the biological deterioration can likely be ascribed to the local environmental conditions in each area [46]. All things considered, the results of this field application were regarded as highly positive [46].

The effectiveness and the aesthetic compatibility of the phosphate treatment have been evaluated in a field study in Bologna (Italy), performed on a mausoleum originally dating back to the XIII century, that was bombed during WWII and rebuilt in the 50s [95]. The base of a column (presumably made of a compact variety of marble, the so-called “*Biancone di Verona*”), affected by cracks and some material loss (but no pulverization or flaking), was selected for the test. The column base was treated with a 3 M DAP solution for 72 h, followed by a limewater poultice for 48 h. After treatment, a general increase in *UPV* (measured across the column base in several directions, so as to obtain a sort of tomographic representation) was found, the increase reaching Δ*UPV* = +1.7 km/s near the edges of the column base [95]. No macroscopic alteration in the visual appearance occurred after treatment, as confirmed by colorimetric measurements carried out in 19 points: in 12 cases the colour change was below the “just noticeable difference” (ΔE* = 2.3 [76]) and only in 3 cases it was above the common acceptability threshold (ΔE* = 5 [66]) [95]. However, it should be noted that, even before the treatment, a great chromatic heterogeneity was present, because of several veins in the type of marble [95]. Some white spots visible after application of the DAP solution (likely containing unreacted DAP) were removed after application of the limewater poultice, rinsing and gently brushing [95]. All things considered, both the efficacy and the aesthetic compatibility of the phosphate treatment were found to be suitable in this study case, which is currently periodically monitored to assess the treatment effects in the long treatment [95].

## 7. Conclusions

The phosphate treatment is based on application of an aqueous solution of a phosphate salt (generally, diammonium hydrogen phosphate, DAP) to induce formation of new calcium phosphate phases (ideally, hydroxyapatite, HAP) inside the micro-cracks and on the surface of the treated substrate. So-formed calcium phosphates have been found to effectively restore mechanical properties of weathered marble and limestone (by re-establishing cohesion among calcite grains) and to significantly increase resistance of marble to dissolution in acidic solutions (thanks to the lower solubility of HAP with respect to calcite).

Compared to alternative products commonly used for marble and limestone conservation (viz., ammonium oxalate and ethyl silicate), the phosphate treatment offers several advantages:

- the DAP solution has low viscosity, so it is able to penetrate deeply into weathered marble (>20 mm) and limestone (>25 mm), while ammonium oxalate is generally affected by low penetration (1–2 mm);
- the phosphate solution causes significant mechanical strengthening after curing for a short time (24–48 h), while curing of ethyl silicate requires more than 6 months;
- the newly formed calcium phosphates do not significantly alter porosity and pore size distribution and leave the treated stone hydrophilic, so that minor changes in water absorption, drying rate and water vapour permeability are experienced. On the contrary, ethyl silicate leaves the treated stone hydrophobic for several months and often causes significant alterations in the pore system;
- as a consequence of the reduced alterations in microstructural and physical properties, stones treated by the phosphate treatment exhibit good durability to heating-cooling cycles, freeze-thaw cycles and salt crystallization cycles; on the contrary, worsened durability was experienced by marble treated with ammonium oxalate and limestone treated with ethyl silicate;
- the phosphate treatment does not cause significant colour change (often below the human eye detection limit and generally below the threshold commonly accepted for conservation treatments); on the contrary, ethyl silicate can cause unacceptable darkening of marble;
- no toxic compound is involved in the phosphate treatment, whereas ethyl silicate is often applied in organic solvents (e.g., white spirit) that can be toxic for human health and the environment;
- because pores are not significantly occluded and the stone remains hydrophilic after treatment, DAP-treated stone can be retreated in the future by either the same treatment or a different one.

The high potential of the phosphate treatment has been confirmed in several field studies carried out on marble and limestone artefacts. In addition to carbonate stones, for which it was originally conceived, the phosphate treatment has recently been explored for a variety of different substrates, such as sandstones, sulphated stones, gypsum stuccoes, concrete, wall paints, archaeological bones and paper, with encouraging results in all cases.

Among the aspects needing further investigation and optimization, future research should be addressed to better elucidate the following points:

- *the use of organic additions or templates to optimize marble surface coverage*: a few calcite grains, presumably having unfavourable crystallographic orientation, were found to remain uncoated by the calcium phosphate deposits, even when ethanol and isopropanol were added to improve the coating formation. Different organic additions and/or the use of templates should be investigated to favour nucleation on the calcite surface.
- *the role of magnesium in the substrate*: even small amounts of magnesium have been found to significantly alter formation of calcium phosphates (possibly leading to formation of magnesium phosphates or magnesium ammonium phosphates), hence the influence of magnesium in the substrate should be systematically investigated and methods to prevent its negative influence should be developed. This will be of interest for several different magnesium-containing substrates, ranging from dolomitic marble to ivory.
- *the effectiveness and the durability of coatings functionalized with nanoparticles*: in addition to combination with nanoTiO_2_ to achieve self-cleaning ability and with nanosilver to achieve anti-fungal activity, further functionalization of the calcium phosphate coatings by nanoparticle addition is worthy of investigation. In the case of silver nanoparticles and strontium-, barium- and silver-substituted HAP for anti-fungal activity, the effectiveness, the compatibility and the durability in real practical applications should also be investigated.
- *the effectiveness and the durability of aluminium phosphates*: preliminary results have indicated the high potential of aluminium phosphates for consolidation of marble, thanks to the very good match in lattice parameters between calcite and AlPO_4_ (the mineral berlinite). Future research should be devoted to further optimize the treatment conditions (in terms of precursors, pH, organic additions, etc.) and to assess the treatment durability.

## Figures and Tables

**Table 1:** Comparison between lattice parameters and crystal structure [25,26], solubility product in water at 25 °C (*K_sp_*) [22,27,28] and dissolution rate in water at pH 5.6 (*R_diss_*) [29,30] of calcite, whewellite and hydroxyapatite. For calcite, two molecules for unit cell are considered to show the match in lattice parameters with hydroxyapatite. For hydroxyapatite, the solubility product is estimated as ~10^−117^ [22] or ~10^−58^ [31], depending on whether the Ca_10_(PO_4_)_6_(OH)_2_ or the Ca_5_(PO_4_)_3_(OH) formula is considered, respectively. (n.a. = not available)

Mineral	a (Å)	b (Å)	c (Å)	Crystal Structure	Solubility Product *K_sp_*	Dissolution Rate *R_diss_* (mol/(cm^2^ s))
Calcite (2×)	9.98	9.98	33.82	Rhombohedral	5 × 10^−9^	~10^−10^
Whewellite	6.29	14.58	10.16	Hexagonal	~10^−9^	n.a.
Hydroxyapatite	9.43	9.42	6.88	Monoclinic	~10^−117^	~10^−14^

**Table 2:** Nomenclature and solubility of CaP phases [22], compared to calcite [27,36], gypsum [8,36] and whewellite [28] (n.a. = not available).

Acronym	Mineral	Formula	Ca/P Ratio	Solubility Product *K_ps_*	Solubility at 25 °C (g/L)
HAP	Hydroxyapatite	Ca_10_(PO_4_)_6_(OH)_2_	1.67	~10^−117^	~0.0003
CDHAP	Calcium-deficient HAP	Ca_10−x_(HPO_4_)_x_(PO_4_)_6−x_ (OH)_2−x_ (0 < x < 1)	1.5–1.67	~10^−85^	~0.0094
ACP	Amorphous calcium phosphate	Ca_x_H_y_(PO_4_)_z_∙nH_2_O (n = 3–4.5, 15–20% H_2_O)	1.2–2.2	~10^−25^–10^−33^	n.a.
β-TCP	β-Tricalcium phosphate (or calcium phosphate tribasic)	β-Ca_3_(PO_4_)_2_	1.5	~10^−29^	~0.0005
OCP	Octacalcium phosphate	Ca_8_H_2_(PO_4_)_6_·5H_2_O	1.33	~10^−97^	~0.0081
DCPD	Dicalcium phosphate dihydrate (or brushite)	CaHPO_4_∙2H_2_O	1.0	~10^−7^	~0.088
DCPA	Dicalcium phosphate anhydrous (or monetite)	CaHPO_4_	1.0	~10^−7^	~0.0048
MCPM	Monocalcium phosphate monohydrate	Ca(HPO_4_)_2_∙H_2_O	0.5	~10^−1^	~18
MCPA	Monocalcium phosphate anhydrous (or calcium phosphate monobasic)	Ca(HPO_4_)_2_	0.5	~10^−1^	~17
-	Calcite	CaCO_3_	-	5 × 10^−9^	~0.014
-	Gypsum	CaSO_4_∙2H_2_O	-	9 × 10^−6^	~2.4
-	Whewellite	CaC_2_O_4_∙H_2_O	-	~10^−9^	n.a.

**Table 3:** Mineralogical composition of the CaP phases formed on calcitic powders, marble and limestone, as a function of the treatment conditions (n.a. = not available, ? = not conclusive identification).

Substrate	Treating Solution	pH	Application Method	Characterization Techniques	CaP	Ref
Calcite powder	10% DAP (24–48 h)	7.8–8.8	Immersion	XRD, FT-IR, μ-Raman	HAP, OCP, brushite	[35]
Compacted calcite powder	nano-Ca(OH), then 5% TAP (14 days)	n.a.	Spraying	XRD	HAP	[4]
Compacted calcite powder	5% *w*/*v* DAP	n.a.	Poultice	μ-Raman	HAP	[61]
White marble	Collagen, then 10 mM CaCl_2_ + 6 mM DAP	n.a.	Dripping	XRD	HAP	[58]
White marble	1 g/L monocalcium phosphate (72 h)	n.a.	Poultice	XRD	HAP	[59]
White marble	Collagen, then 5 g/L TAP and 0.6 g/L NH_4_F (48 h)	n.a.	Poultice	XRD	Fluorapatite	[54]
Carrara marble (fresh)	1 M DAP (24 h)	8	Immersion	GID	HAP	[34]
Carrara marble (fresh)	1 M DAP + 1 mM CaCl_2_ (24 h)	8	Immersion	GID	HAP, OCP	[34]
Carrara marble (artificially weathered)	3 M DAP (48 h)	n.a.	Brushing (15 times)	μ-Raman	ACP? MCPA? Residual DAP	[43]
Carrara marble (artificially weathered)	3 M DAP (48 h), then limewater	n.a.	Brushing (15 times)	μ-Raman	OCP, TCP?	[43]
Carrara marble (artificially weathered)	3 M DAP + 1 mM CaCl_2_ (48 h), then limewater	n.a.	Brushing (15 times)	μ-Raman	OCP, TCP?	[43]
Carrara marble (artificially weathered)	3 M DAP + 3 mM CaCl_2_ (48 h), then limewater	n.a.	Brushing (15 times)	μ-Raman	OCP	[43]
Carrara marble (naturally weathered)	3 M DAP (48 h), then limewater (24 h)	n.a.	Brushing (15 times)	μ-Raman	HAP	[43]
Carrara marble (fresh)	1 M DAP + 1 mM CaCl_2_ (24 h)	8	Immersion	GID	HAP, OCP	[38]
Carrara marble (fresh)	0.1 M DAP + 0.1 mM CaCl_2_ in 10 vol % ethanol (24 h)	8	Immersion	GID	OCP	[38]
Carrara marble (fresh)	0.1 M DAP + 0.1 mM CaCl_2_ in 10 vol % isopropanol (24 h)	8	Immersion	GID	OCP	[38]
Carrara marble (artificially weathered)	3 M DAP (48 h), followed by limewater poultice	n.a.	Brushing (8 times)	Raman	HAP (OCP?)	[45]
Carrara marble (artificially weathered)	0.1 M DAP + 0.1 mM CaCl_2_ in 10 vol % ethanol (24 h), twice	n.a.	Brushing (5 times)	FT-IR	OCP	[44]
Carrara marble (artificially weathered)	0.1 M DAP + 0.1 mM CaCl_2_ in 10 vol % isopropanol (24 h), twice	n.a.	Brushing (8 times)	FT-IR	OCP	[44]
Carrara marble (artificially weathered)	1 M DAP + 1 mM CaCl_2_ (24 h)	n.a.	Brushing (5 times)	FT-IR	HAP, OCP	[44]
Carrara marble (artificially weathered)	3 M DAP (48 h), followed by limewater poultice	n.a.	Brushing (8 times)	FT-IR	HAP, OCP?	[44]
Indiana limestone (artificially weathered)	1 M DAP (48 h)	8	Capillarity	EBSD	HAP, OCP, CDHAP, MCPM	[1]
Lecce stone (fresh)	5% DAP (4–8–17 h)	8	Poultice	XRD, FT-IR	HAP	[3]
Lecce stone (fresh)	5% ADP (4–8 h)	5.6–6	Poultice	XRD, FT-IR	HAP, brushite	[3]
Lecce stone (fresh)	5% ADP (4–8–17 h)	7–8	Poultice	XRD, FT-IR	HAP	[3]
Tuffeau de Maastricht (fresh)	5% DAP (4–8–17 h)	8	Poultice	XRD, FT-IR	HAP	[3]
Tuffeau de Maastricht (fresh)	5% ADP	5.6–6	Poultice	XRD, FT-IR	brushite	[3]
Tuffeau de Maastricht (fresh)	5% ADP	7–8	Poultice	XRD, FT-IR	HAP	[3]
Globigerina limestone (artificially weathered)	3 M DAP (48 h), then limewater	n.a.	Brushing (10 or 20 times), Poultice, Immersion	FT-IR	HAP	[42]
Arenisca Ronda (artificially sulphated)	3 M DAP (1 h)	n.a.	Poultice	XRD	HAP, brushite, TCP?	[9]
Marlstone (naturally weathered)	1 M DAP (3 h)	n.a.	Poultice	XRD, FT-IR	HAP	[46]

**Table 4:** Consolidating ability of the phosphate treatment applied to marble (n.a. = not available; D = diameter; H = height; UW = unweathered; W = weathered; UT = untreated; TR = treated; w,_STT_ = weight loss after scotch tape test; σ_c_ = compressive strength; F_c_ = compression force for a given cross section; UPV = ultrasonic pulse velocity; *E_d_* = dynamic elastic modulus; * multiple values are reported for samples preliminarily decayed to two different levels; ** multiple values are reported for two different directions, parallel and perpendicular to marble foliation).

Substrate	Specimen	Treating Solution	Application Method	Penetration Depth	Consolidating Action	Ref
Compacted calcite powder	n.a.	nano-Ca(OH)_2_, then 5% TAP (2 weeks)	Spraying	n.a.	- UT: w,_STT_ = 19.4 mg/cm^2^, σ_c_ < 0.05 MPa - TR: w,_STT_ = 0.05 mg/cm^2^, σ_c_ = 4.5 MPa	[4]
Compacted calcite powder	Cylinders (D = 39.8 mm, H = 80 mm)	Collagen, then 10 mM CaCl_2_ + 6 mM DAP	Dripping	n.a.	- UT: F_c_ = 200 N - TR: F_c_ = 300 N	[58]
Compacted calcite powder	Cylinders (D = 15 mm, H = 4 mm)	5% *w*/*v* DAP	Poultice	HAP formation: 2 mm (μ-Raman)	n.a.	[61]
Compacted calcite powder	Cylinders (D = 15 mm, H = 4 mm)	5% *w*/*v* DAP, then 5% *w*/*v* AmOx	Poultice	HAP formation: 2.5 mm (μ-Raman)	n.a.	[61]
Carrara marble (naturally weathered)	n.a.	5% *w*/*v* DAP, then 5% *w*/*v* AmOx	Poultice	HAP formation: 2.5 mm (μ-Raman)	n.a.	[61]
Carrara marble (artificially weathered)	Slabs (65 × 65 × 20 mm^3^)	3 M DAP (48 h), followed by limewater poultice	Brushing (15 times)	- DAP solution: at least 20 mm - CaP formation: at least 4 mm (abrasion resistance)	- UW,UT: UPV = 2.5 km/s - W,UT: UPV = 0.8 km/s - W,TR: UPV = 4.0 km/s	[43]
White marble (naturally weathered)	Slab (120 × 90 × 30 mm^3^)	3 M DAP (48 h), followed by limewater poultice	Brushing (15 times)	n.a.	UPV increase from ~88% to ~98% of the maximum UPV value	[43]
Carrara marble (artificially weathered)	Slabs (30 × 30 × 20 mm^3^)	3 M DAP (48 h), followed by limewater poultice	Brushing (8 times)	n.a.	- UW,UT: UPV = 2.9 km/s - W,UT: UPV = 0.7 km/s - W,TR: UPV = 3.1 km/s	[45]
Carrara marble (artificially weathered)	Slabs (30 × 30 × 20 mm^3^)	3 M DAP (48 h), followed by limewater poultice (24 h), then nanoTiO_2_	Brushing (8 times)	n.a.	- UW,UT: UPV = 2.9 km/s - W,UT: UPV = 0.7 km/s - W,TR: UPV = 3.0 km/s	[45]
Carrara marble (artificially weathered)	Slabs (30 × 30 × 20 mm^3^)	3 M DAP with nanoTiO_2_ (48 h), followed by limewater poultice (24 h)	Brushing (8 times)	n.a.	- UW,UT: UPV = 2.9 km/s - W,UT: UPV = 0.7 km/s - W,TR: UPV = 3.1 km/s	[45]
Carrara marble (artificially weathered)	Slabs (30 × 30 × 20 mm^3^)	0.1 M DAP+0.1 mM CaCl_2_ + 0.5 wt % ethanol (24 h), twice (the second time without ethanol)	Immersion	n.a.	- UW,UT: UPV = 3.2 km/s - W,UT: UPV = 0.6 km/s - W,TR: UPV = 2.9 km/s	[64]
Carrara marble (artificially weathered)	Slabs (30 × 30 × 20 mm^3^)	3 M DAP (48 h), followed by limewater poultice	Brushing (15 times)	n.a.	- UW,UT: UPV = 3.2 km/s - W,UT: UPV = 0.6 km/s - W,TR: UPV = 2.2 km/s	[64]
Carrara marble (artificially weathered)	Slabs (50 × 50 × 10 mm^3^)	0.1 M DAP + 0.1 mM CaCl_2_ in 10 vol % ethanol (24 h)	Vacuum saturation	n.a.	- W,UT: *E_d_* = 61% of the initial *E_d_* - W,TR: *E_d_* = 88% of the initial *E_d_* (single application) - W,TR: *E_d_* = 113% of the initial *E_d_* (double application)	[39]
Carrara marble (artificially weathered)	Slabs (50 × 50 × 10 mm^3^)	1 M DAP + 1 mM CaCl_2_ (24 h)	Vacuum saturation	n.a.	- UW,UT: *E_d_* = 68–72 GPa * - W,UT: *E_d_* = 38–46 GPa * - W,TR: *E_d_* = 97–98 GPa *	[38]
Carrara marble (artificially weathered)	Slabs (50 × 50 × 10 mm^3^)	0.1 M DAP + 0.1 mM CaCl_2_ in 10 vol % ethanol (24 h)	Vacuum saturation	n.a.	- UW,UT: *E_d_* = 68–72 GPa * - W,UT: *E_d_* = 38–46 GPa * - W,TR: *E_d_* = 61–68 GPa (single application) * - W,TR: *E_d_* = 61–76 GPa * (double application) *	[38]
Carrara marble (artificially weathered)	Slabs (50 × 50 × 10 mm^3^)	0.1 M DAP + 0.1 mM CaCl_2_ in 10 vol % isopropanol (24 h)	Vacuum saturation	n.a.	- UW,UT: *E_d_* = 68–72 GPa * - W,UT: *E_d_* = 38–46 GPa * - W,TR: *E_d_* = 58–60 GPa (single application) * - W,TR: *E_d_* = 67–80 GPa * (double application) *	[38]
Carrara marble (artificially weathered)	Cylinders (D = 15 mm, H = 50 mm)	0.1 M DAP + 0.1 mM CaCl_2_ in 10 vol % ethanol (24 h), twice	Brushing (5 times)	DAP solution: at least 7.5 mm	- UW,UT: *E_d_* = 53–76 GPa ** - W,UT: *E_d_* = 14–24 GPa ** - W,TR: *E_d_* = 64–93 GPa **	[44]
Carrara marble (artificially weathered)	Slabs (400 × 100 × 20 mm^3^)	0.1 M DAP + 0.1 mM CaCl_2_ in 10 vol % ethanol (24 h), twice	Brushing (8 times)	DAP solution: at least 20 mm	- UW,UT: *E_d_* = 58–60 GPa ** - W,UT: *E_d_* = 16–19 GPa ** - W,TR: *E_d_* = 22–32 GPa **	[44]
Carrara marble (artificially weathered)	Cylinders (D = 15 mm, H = 50 mm)	0.1 M DAP + 0.1 mM CaCl_2_ in 10 vol % isopropanol (24 h), twice	Brushing (5 times)	DAP solution: at least 7.5 mm	- UW,UT: *E_d_* = 53–76 GPa ** - W,UT: *E_d_* = 14–24 GPa ** - W,TR: *E_d_* = 70–91 GPa **	[44]
Carrara marble (artificially weathered)	Slabs (400 × 100 × 20 mm^3^)	0.1 M DAP + 0.1 mM CaCl_2_ in 10 vol % isopropanol (24 h), twice	Brushing (8 times)	DAP solution: at least 20 mm	- UW,UT: *E_d_* = 58–60 GPa ** - W,UT: *E_d_* = 16–19 GPa ** - W,TR: *E_d_* = 27–37 GPa **	[44]
Carrara marble (artificially weathered)	Cylinders (D = 15 mm, H = 50 mm)	1 M DAP + 1 mM CaCl_2_ (24 h)	Brushing (5 times)	DAP solution: at least 7.5 mm	- UW,UT: *E_d_* = 53–76 GPa ** - W,UT: *E_d_* = 14–24 GPa ** - W,TR: *E_d_* = 110–112 GPa **	[44]
Carrara marble (artificially weathered)	Slabs (400 × 100 × 20 mm^3^)	1 M DAP + 1 mM CaCl_2_ (24 h)	Brushing (7 times)	DAP solution: at least 20 mm	- UW,UT: *E_d_* = 58–60 GPa ** - W,UT: *E_d_* = 16–19 GPa ** - W,TR: *E_d_* = 52–59 GPa **	[44]
Carrara marble (artificially weathered)	Cylinders (D = 15 mm, H = 50 mm)	3 M DAP (24 h), followed by limewater poultice	Brushing (5 times)	DAP solution: at least 7.5 mm	- UW,UT: *E_d_* = 53–76 GPa ** - W,UT: *E_d_* = 14–24 GPa ** - W,TR: *E_d_* = 79–93 GPa **	[44]
Carrara marble (artificially weathered)	Slabs (400 × 100 × 20 mm^3^)	3 M DAP (24 h), followed by limewater poultice	Brushing (4 times)	DAP solution: at least 20 mm	- UW,UT: *E_d_* = 58–60 GPa ** - W,UT: *E_d_* = 16–19 GPa ** - W,TR: *E_d_* = 62–69 GPa **	[44]

**Table 5:** Colour change in marble after the phosphate treatment (L* = black ÷ white, a* = green ÷ red, b* = blue ÷ yellow, ΔE* = colour change, n.a. = not available).

Substrate	Treating Solution	Application Method	ΔL*	Δa*	Δb*	ΔE*	Ref
Carrara marble (fresh)	0.1 M DAP + 0.1 mM CaCl_2_ in 0.5 wt % ethanol (24 h), twice (the second time without ethanol)	Immersion	n.a.	n.a.	n.a.	0.4	[63]
Carrara marble (fresh)	3 M DAP (48 h), followed by limewater poultice	Brushing (8 times)	n.a.	n.a.	n.a.	0.6	[63]
White marble (fresh)	1 g/L monocalcium phosphate	Poultice	+1.7	−1.2	−0.8	2.2	[59]
White marble (fresh)	0.1 g/L Type I collagen + 5 g/L TAP + 0.6 g/L NH_4_F	Poultice	+1.5	−1.4	−0.7	2.1	[54]
Carrara marble (artificially weathered)	0.1 M DAP+0.1 mM CaCl_2_ in 0.5 wt % ethanol (24 h), twice (the second time without ethanol)	Immersion	n.a.	n.a.	n.a.	1.1	[64]
Carrara marble (artificially weathered)	3 M DAP (48 h), followed by limewater poultice	Brushing (15 times)	n.a.	n.a.	n.a.	1.5	[64]
Carrara marble (artificially weathered)	3 M DAP (48 h), followed by limewater poultice	Brushing (15 times)	−1.9	−0.1	−0.4	1.9	[43]
White marble (naturally weathered)	3 M DAP (48 h), followed by limewater poultice	Brushing (15 times)	+2.1	−0.1	−1.7	2.7	[43]
Carrara marble (artificially weathered)	1 M DAP + 1 mM CaCl_2_ (24 h)	Vacuum saturation	−2.0	−0.2	−0.6	2.1	[38]
Carrara marble (artificially weathered)	0.1 M DAP + 0.1 mM CaCl_2_ in 10 vol % ethanol (24 h)	Vacuum saturation	−1.4	+0.0	−0.1	1.4	[38]
Carrara marble (artificially weathered)	0.1 M DAP + 0.1 mM CaCl_2_ in 10 vol % ethanol (24 h), twice	Vacuum saturation	−1.5	+0.0	−0.6	1.6	[38]
Carrara marble (artificially weathered)	0.1 M DAP + 0.1 mM CaCl_2_ in 10 vol % isopropanol (24 h)	Vacuum saturation	−1.4	−0.4	−0.7	1.6	[38]
Carrara marble (artificially weathered)	0.1 M DAP + 0.1 mM CaCl_2_ in 10 vol % isopropanol (24 h), twice	Vacuum saturation	−2.0	−0.3	−1.0	2.2	[38]
Carrara marble (artificially weathered)	3 M DAP (48 h), followed by limewater poultice	Brushing (8 times)	+1.8	−0.3	−2.2	2.2	[45]
Carrara marble (artificially weathered)	3 M DAP (48 h), followed by limewater poultice (24 h), then nanoTiO_2_	Brushing (8 times)	+1.2	0.2	−1.5	1.2	[45]
Carrara marble (artificially weathered)	3 M DAP with nanoTiO_2_ (48 h), followed by limewater poultice (24 h)	Brushing (8 times)	−0.2	−0.3	−1.7	0.6	[45]
White marble (artificially sulphated)	0.02 M DAP at pH 9.2 (5 days)	Poultice	n.a.	n.a.	n.a.	1.5	[8]

**Table 6:** Consolidating ability of the phosphate treatment applied to limestone (D = diameter; H = height; UW = unweathered; W = weathered; UT = untreated; TR = treated; *E_d_* = dynamic elastic modulus; σ_t_ = tensile strength; σ_c_ = compressive strength; MDR = micro-drilling resistance in the first 10 mm from the treated surface; HD = hardness; w,_STT_ = weight loss after the scotch tape test).

Substrate	Specimen	Treating Solution	Application Method	Penetration Depth	Consolidating Action	Ref
Indiana limestone (artificially weathered)	Cylinders (D = 20 mm, H = 50 mm)	1 M DAP (48 h)	Immersion	CaP formation: 20 mm (EBSD, MIP)	- UW,UT: *E_d_* = 35 GPa, Δσ_t_ = 4.8 MPa - W,UT: *E_d_* = 19 GPa, Δσ_t_ = 3.3 MPa - W,TR: *E_d_* = 36 GPa, Δσ_t_ = 4.1 MPa	[1]
Indiana limestone (artificially weathered)	Cylinders (D = 20 mm, H = 50 mm)	1 M DAP (48 h)	Brushing	n.a.	- UW,UT: *E_d_* = 35 GPa, Δσ_t_ = 4.8 MPa - W,UT: *E_d_* = 23 GPa, Δσ_t_ = 3.5 MPa - W,TR: *E_d_* = 40 GPa, Δσ_t_ = 4.1 MPa	[79]
Indiana limestone (artificially weathered)	Cubes (50 mm side)	1 M DAP + 1 mM CaCl_2_ (48 h)	Capillarity	n.a.	- UW,UT: *E_d_* = 35 GPa - W,UT: *E_d_* = 22 GPa W,TR: *E_d_* = 46 GPa	[48]
Globigerina limestone (artificially weathered)	Cylinders (D = 20 mm, H = 50 mm)	3 M DAP, followed by limewater poultice	Brushing (10 times)	- DAP solution: 8–9 mm - HAP formation: at least 7.5 mm (abrasion resistance), at least 10 mm (FT-IR)	- UW,UT: *E_d_* = 16 GPa, Δσ_t_ = 3.0 MPa - W,UT: *E_d_* = 11 GPa, Δσ_t_ = 2.7 MPa - W,TR: *E_d_* = 16 GPa, Δσ_t_ = 3.4 MPa	[42]
Globigerina limestone (artificially weathered)	Cylinders (D = 20 mm, H = 50 mm)	3 M DAP, followed by limewater poultice	Brushing (20 times)	- DAP solution: 8–9 mm - HAP formation: at least 7.5 mm (abrasion resistance), at least 10 mm (FT-IR)	- UW,UT: *E_d_* = 16 GPa, Δσ_t_ = 3.0 MPa - W,UT: *E_d_* = 11 GPa, Δσ_t_ = 2.7 MPa - W,TR: *E_d_* = 16 GPa, Δσ_t_ = 3.2 MPa	[42]
Globigerina limestone (artificially weathered)	Cylinders (D = 20 mm, H = 50 mm)	3 M DAP, followed by limewater poultice	Poultice	- DAP solution: 25 mm - HAP formation: at least 7.5 mm (abrasion resistance), at least 10 mm (FT-IR)	- UW,UT: *E_d_* = 16 GPa, Δσ_t_ = 3.0 MPa - W,UT: *E_d_* = 11 GPa, Δσ_t_ = 2.7 MPa - W,TR: *E_d_* = 16 GPa, Δσ_t_ = 3.4 MPa	[42]
Globigerina limestone (artificially weathered)	Cylinders (D = 20 mm, H = 50 mm)	3 M DAP, followed by limewater poultice	Immersion	- DAP solution: 25 mm - HAP formation: at least 7.5 mm (abrasion resistance), at least 10 mm (FT-IR)	- UW,UT: *E_d_* = 16 GPa, Δσ_t_ = 3.0 MPa - W,UT: *E_d_* = 11 GPa, Δσ_t_ = 2.7 MPa - W,TR: *E_d_* = 17 GPa, Δσ_t_ = 3.5 MPa	[42]
Lecce stone (fresh)	Slabs (50 × 50 × 20 mm^3^)	5% DAP solution for 4–8–17 h	Poultice	HAP formation: at least 10 mm (MDR)	- UT: MDR = 33 N - TR: MDR = 38–39 N	[3]
Lecce stone (fresh)	Slabs (50 × 50 × 20 mm^3^)	5% ADP solution for 4–8 h	Poultice	HAP formation: at least 10 mm (MDR)	- UT: MDR = 33 N - TR: MDR = 37–39 N	[3]
Lecce stone (fresh)	Slabs (50 × 50 × 20 mm^3^)	5% ADP solution at pH 8 for 4–8–17 h	Poultice	HAP formation: at least 10 mm (MDR)	- UT: MDR = 33 N - TR: MDR = 38–41 N	[3]
Tuffeau de Maastricht (fresh)	Slabs (50 × 50 × 20 mm^3^)	5% DAP solution for 4–8–17 h	Poultice	HAP formation: at least 10 mm (MDR)	- UT: MDR = 3.1 N - TR: MDR = 3.0–3.8 N	[3]
Tuffeau de Maastricht (fresh)	Slabs (50 × 50 × 20 mm^3^)	5% ADP solution for 4–8 h	Poultice	brushite formation: at least 10 mm (MDR)	- UT: MDR = 3.1 N - TR: MDR = 4.1–4.3 N	[3]
Tuffeau de Maastricht (fresh)	Slabs (50 × 50 × 20 mm^3^)	5% ADP solution at pH 8 for 4–8–17 h	Poultice	HAP formation: at least 10 mm (MDR)	- UT: MDR = 3.1 N - TR: MDR = 4.1–4.8 N	[3]
Compacted limestone powder	Cylinders (D = 32 mm, H = n.a.)	1 M DAP (3 months)	Brushing (until refusal)	n.a.	- UT: HD = 60 HD, σ_c_ = 77 MPa, w,_STT_ = 1.2 mg/cm^2^ - TR: HD = 83 HD, σ_c_ = 177 MPa, w,_STT_ = 0.01 mg/cm^2^	[57]
Compacted limestone powder	Cylinders (D = 32 mm, H = n.a.)	1 M DAP + 0.1 M CTAB (3 months)	Brushing (until refusal)	n.a.	- UT: HD = 60 HD, σ_c_ = 77 MPa, w,_STT_ = 1.2 mg/cm^2^ - TR: HD = 84 HD, σ_c_ = 180 MPa, w,_STT_ = 0.01 mg/cm^2^	[57]
Arenisca Ronda (artificially sulphated)	Slabs (25 × 20 × 10 mm^3^)	3 M DAP for 60 min	Poultice	CaP formation: 3.5 mm	- W,UT: MDR = 1.27 N/mm - W,TR: MDR = 1.51 N/mm	[9]

**Table 7:** Colour change of limestone after the phosphate treatment (L* = black ÷white, a* = green ÷ red, b* = blue ÷ yellow, ΔE* = colour change, n.a. = not available; * in this stone, colour differences up to ΔE* = 11.5 were measured among different untreated samples).

Substrate	Treating Solution	Application Method	ΔL*	Δa*	Δb*	ΔE*	Ref
Indiana limestone (artificially weathered)	1 M DAP	Immersion	n.a.	n.a.	n.a.	7.6 *	[1]
Lecce stone (fresh)	5% DAP solution for 4–8–17 h	Poultice	−2.5 to −0.5	0 to +0.4	−2.8 to +0.6	2.1 to 2.9	[3]
Lecce stone (fresh)	5% ADP solution for 4–8 h	Poultice	−0.7 to 0	+0.9 to +1.1	−1.7 to −1.3	1.6 to 2.2	[3]
Lecce stone (fresh)	5% ADP solution at pH 8 for 4–8–17 h	Poultice	−1.2 to +3.7	0.1 to 0.6	−4.3 to −0.1	1.2 to 5.7	[3]
Tuffeau de Maastricht (fresh)	5% DAP solution for 4–8–17 h	Poultice	+0.3 to +0.5	−0.1 to 0	−1.7 to −0.9	0.9 to 1.7	[3]
Tuffeau de Maastricht (fresh)	5% ADP solution for 4–8 h	Poultice	+0.6 to +2.4	−0.1 to 0	−3.2 to +0.3	0.7 to 4.0	[3]
Tuffeau de Maastricht (fresh)	5% ADP solution at pH 8 for 4–8–17 h	Poultice	+0.1 to +3.1	+0.2	−4.0 to −1.0	1.1 to 5.1	[3]
Globigerina limestone (artificially weathered)	3 M DAP, followed by limewater poultice	Brushing (10 times)	−0.7	+1.9	+1.9	2.8	[42]
Globigerina limestone (artificially weathered)	3 M DAP, followed by limewater poultice	Brushing (20 times)	−3.3	+1.1	0.0	3.5	[42]
Globigerina limestone (artificially weathered)	3 M DAP, followed by limewater poultice	Poultice	−0.2	0.0	−3.4	3.4	[42]
Globigerina limestone (artificially weathered)	3 M DAP, followed by limewater poultice	Immersion	−0.5	0.0	−2.2	2.2	[42]
Globigerina limestone (artificially weathered)	3 M DAP, followed by limewater poultice	Brushing (10 times)	−4.0	+1.5	+1.3	4.4	[49]
Compacted limestone powder	1 M DAP (3 months)	Brushing (until refusal)	n.a.	n.a.	n.a.	2.3	[57]
Compacted limestone powder	1 M DAP + 0.1 M CTAB (3 months)	Brushing (until refusal)	n.a.	n.a.	n.a.	3.3	[57]
Arenisca Ronda (artificially sulphated)	3 M DAP for 60 min	Poultice	n.a.	n.a.	n.a.	<3	[9]
